# Additive Manufacturing of Lightweight Space Mirrors: A Review of Structural Design, Optical Fabrication, and Performance

**DOI:** 10.3390/s26216698

**Published:** 2026-10-23

**Authors:** Donghui Yu, Zongxuan Li, Jiuxin Ning, Zhen Wen, Shuhui Ren, Qingya Li, Jiangtao Yu, Jiakun Xu

**Affiliations:** 1 Changchun Institute of Optics, Fine Mechanics and Physics, Chinese Academy of Sciences, Changchun 130033, China; yudonghui22@mails.ucas.ac.cn (D.Y.);; 2 University of Chinese Academy of Sciences, Beijing 100049, China; 3 Key Laboratory of Space-Based Dynamic and Rapid Optical Imaging Technology, Chinese Academy of Sciences, Changchun 130033, China

**Keywords:** additive manufacturing, lightweight mirror, mirror materials, ultra-precision machining, surface finishing

## Abstract

As space remote sensing technology continuously evolves toward large apertures and high resolutions, optical systems impose increasingly stringent requirements on the mass, specific stiffness, and surface figure stability of mirrors. Leveraging unparalleled design freedom, additive manufacturing (AM) technology enables the physical fabrication of complex architectures—such as topology optimization (TO), lattices, and Voronoi structures—thereby breaking through the limitations of traditional lightweight mirror designs. This review first introduces the relevant manufacturing technologies for AM space mirrors and analyzes the characteristics of metallic and ceramic materials suitable for AM, with a particular focus on the advantages and limitations of the two most widely applied core materials: AlSi10Mg and silicon carbide (SiC). Subsequently, it reviews the state of the art in AM space mirrors, organized according to their structural typologies. Furthermore, it delves into the critical post-processing technologies required to achieve high-precision surface figures and summarizes the prevailing challenges confronting AM mirrors. Finally, it outlines future research directions for AM mirrors.

## 1. Introduction

Driven by the rapid advancement of space remote sensing and aerospace technologies, large apertures, high resolutions, and wide fields of view have emerged as the core requirements for next-generation space optical systems [1]. As the core components of these systems, mirrors directly dictate the ultimate imaging quality throughout ground-based fabrication, launch overloads, and on-orbit environments; thus, their lightweight ratio, surface figure accuracy, and thermo-mechanical stability are of paramount importance [2]. Achieving an exceptionally high lightweight ratio while suppressing gravity-induced deformation to maintain nanoscale surface figure accuracy remains a formidable challenge in contemporary optomechanical design [3].

Traditional lightweight mirrors predominantly rely on subtractive manufacturing. However, constrained by tool path accessibility and machining allowances, these methods struggle to fabricate complex internal honeycombs or closed sandwich architectures. This limitation not only impedes further improvements in the lightweight ratio but also poses significant challenges when scaling to large apertures and ultra-thin profiles. Under such conditions, conventional processes are highly susceptible to residual stress release and machining chatter, severely hindering substantial breakthroughs in surface figure accuracy. Furthermore, traditional assembled optomechanical systems are prone to thermal stress mismatch under extreme temperature fluctuations.

**Figure 1:**
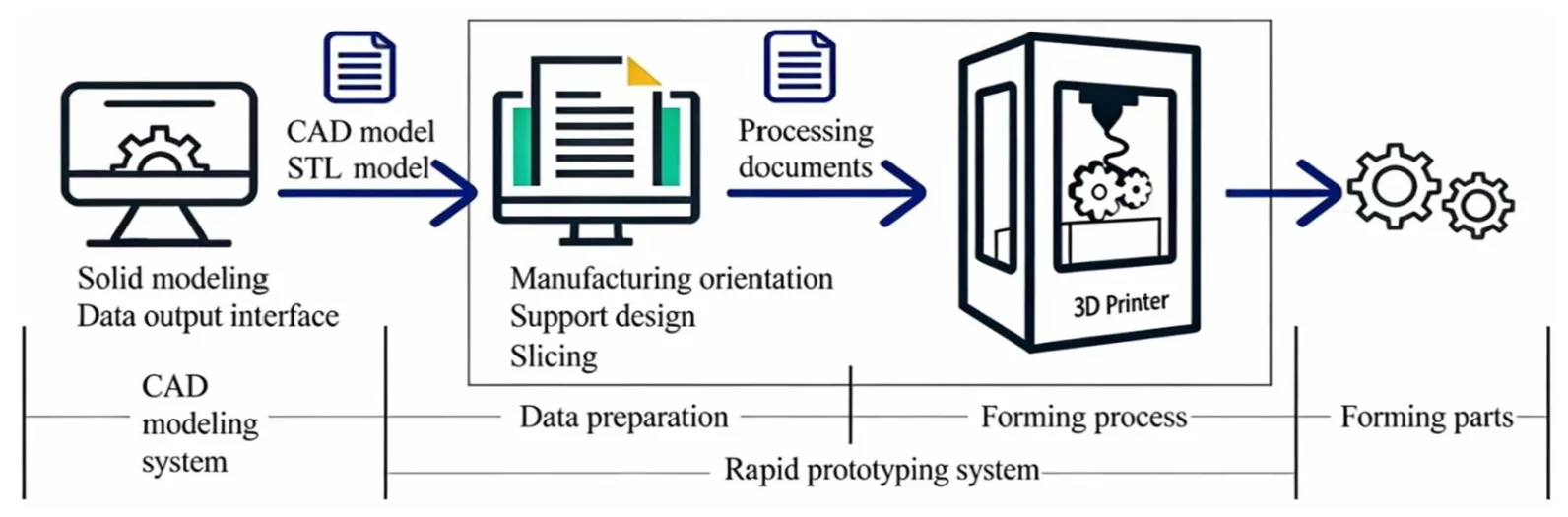
AM process flow [4].

The advent of AM (also known as 3D printing) has introduced a novel paradigm for the design of space mirrors, with its production workflow illustrated in Figure 1 [4]. Utilizing layer-by-layer deposition, AM facilitates the fabrication of complex architectures that transcend traditional geometric constraints, including TO, lattice, and Voronoi structures. More importantly, AM enables monolithic optomechanical design, which significantly enhances the thermal stability of the system by minimizing the number of discrete connecting components [5]. Nevertheless, the implementation of AM mirrors in optical applications presents unique challenges, notably the print-through effect induced by internal support structures and the exacerbated surface scattering caused by microscopic porosity inherent to powder sintering processes. Consequently, optimizing internal lightweight configurations and integrating advanced post-processing techniques—such as ultra-precision polishing and surface modification—have emerged as crucial steps for realizing high-performance AM mirrors.

Unlike previous reviews focusing primarily on individual materials or fabrication routes, this work establishes an integrated framework linking AM process selection, mirror materials, lightweight structural architectures, post-processing strategies, and experimentally demonstrated optical performance. Particular attention is paid to the relationships between structural topology, manufacturability, surface quality, and system-level optical stability.

The remainder of this paper is organized as follows. Section 2 introduces the fundamental technologies associated with the fabrication of AM space mirrors, encompassing AM processing methods, material characteristics, and technological roadmaps. Section 3 provides a systematic review of the latest research progress in AM space mirrors, categorizing them into five distinct lightweight architectures based on their design methodologies, and elaborating on the specific design features of each. Section 4 delves into the critical post-processing techniques essential for achieving high-precision surface figures. Finally, Section 5 and Section 6 summarize the prevailing technical challenges in AM mirror fabrication and propose potential research directions for future development, serving as a reference for researchers in this field.

## 2. Technical Principles of AM for Space Mirrors

### 2.1. Space Mirror AM Technology

AM is a manufacturing technology that fabricates three-dimensional components layer by layer based on digital model data [6]. According to the classification established by the American Society for Testing and Materials (ASTM), AM processes can be categorized into seven major groups: binder jetting (BJT), directed energy deposition (DED), material extrusion (MEX), material jetting (MJT), powder bed fusion (PBF), sheet lamination (SHL), and vat photopolymerization (VPP) [7]. Among these processes, VPP mainly includes stereolithography (SLA) and digital light processing (DLP), while PBF can be further classified into selective laser melting (SLM, also termed L-PBF) and selective laser sintering (SLS).

For the fabrication of optical mirrors, the commonly employed AM technologies mainly include SLM, SLS, SLA, DLP, and electron beam melting (EBM). To provide guidance for researchers in selecting appropriate AM techniques according to specific application requirements, the typical materials, advantages, and limitations of these AM processes applicable to mirror fabrication are compared and summarized in Table 1.

#### 2.1.1. Manufacturing Techniques for Metal Mirrors

**Figure 2:**
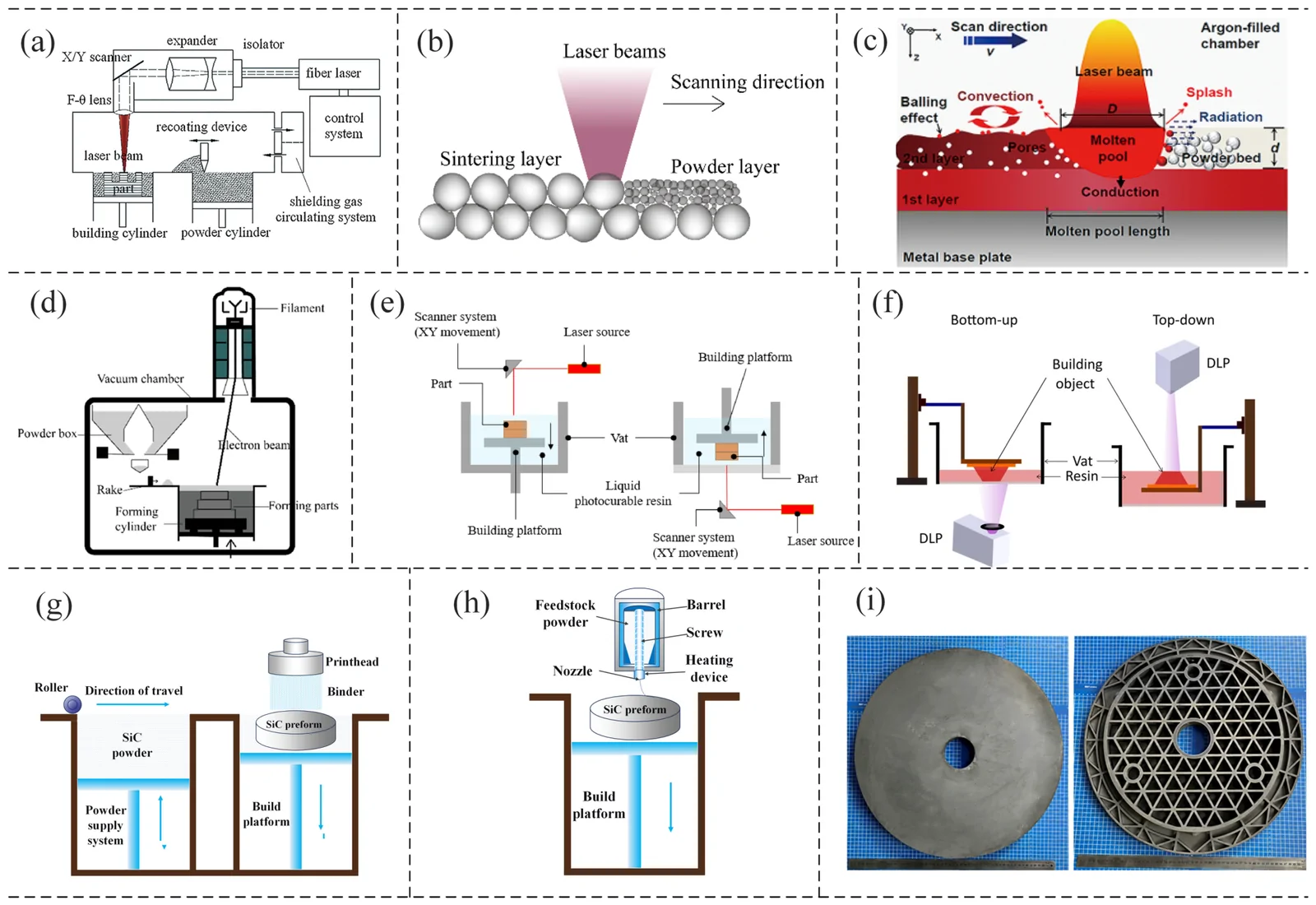
Schematic illustrations of representative AM processes: (**a**) PBF [17]. (**b**) SLS [4]. (**c**) SLM [17]. (**d**) EBM [4]. (**e**) SLA [18]. (**f**) DLP: Bottom-up configuration (left) and Top-down configuration (right) [19]. (**g**) BJT and (**h**) MEX [20]. (**i**) A 500 mm SiC preform: mirror surface (left) and lightweight back structure (right) [21].

In the field of metal mirror fabrication, SLM is considered one of the most promising AM technologies. Unlike early SLS, which primarily relies on solid-state sintering or partial melting between powder particles for material consolidation [16], SLM utilizes a high-energy laser beam to selectively melt metal powders and forms dense metallic structures through rapid solidification of the molten pool. Figure 2a–c illustrates the principle of PBF and the differences in the melting mechanisms between SLM and SLS. As can be observed, SLM-fabricated metallic components generally exhibit high relative density and good metallurgical bonding. However, process-induced defects, including lack-of-fusion (LoF) pores, gas pores, and keyhole defects, may still occur due to variations in laser energy input, scanning strategies, and powder characteristics. Therefore, achieving a highly dense and defect-controlled material state through optimization of process parameters and post-processing treatments is essential for subsequent optical finishing. Such improvements can effectively reduce the risk of surface imperfections and optical scattering caused by internal defects during the polishing process.

In contrast, although EBM offers advantages in processing high-melting-point materials like titanium alloys within a vacuum environment and boasts high energy efficiency [22], its inherent process characteristics result in higher surface roughness and a susceptibility to internal porosity in the as-built components [23,24]. The schematic of EBM is shown in Figure 2d. Although EBM-fabricated titanium alloys typically exhibit higher initial surface roughness, recent studies demonstrate that by optimizing post-processing techniques (such as increasing polishing pressure), EBM-printed Ti64 mirrors can achieve a surface roughness of 2 nm root-mean-square (RMS) and a surface form error of 28 nm RMS. This level of precision is fully adequate for application requirements spanning from visible to infrared wavelengths [25]. Nevertheless, among the metallic AM processes reported for mirror fabrication, SLM has been the most widely investigated [17].

#### 2.1.2. Manufacturing Techniques for Ceramic Mirrors

For high-performance ceramic mirrors such as SiC, fabrication primarily relies on four processes: VPP, SLS, BJT, and MEX [20], with their schematics depicted in Figure 2e–h. The current well-established roadmap involves fabricating a green body via VPP (including SLA and DLP), followed by reactive melt infiltration (RMI) sintering. By utilizing ultraviolet light to cure a ceramic-loaded photosensitive resin slurry layer-by-layer, VPP enables the fabrication of exceptionally complex lightweight architectures with micrometer-level precision [18,19,26]. Subsequently, the as-printed green bodies undergo debinding and densification sintering [27], ultimately transforming into highly densified reaction-bonded silicon carbide (RB-SiC). This methodology has been successfully applied to the manufacture of large-aperture space optics; for instance, W. Li’s team successfully fabricated a SiC mirror blank with a 500 mm aperture utilizing VPP coupled with RMI technology [21], as illustrated in Figure 2i.

In comparison, although SLS is an earlier developed technology, the cyclic thermal stresses inherent to its layer-by-layer sintering process render the preforms susceptible to warpage and internal defects, thereby restricting the fabrication of large-scale components. The BJT technology offers the advantages of being completely support-free, highly efficient, and capable of fabricating large-scale mirrors with complex closed-back structures. However, the printed green parts exhibit high porosity and typically rely on complex densification processes, such as liquid silicon infiltration (LSI), to form RB-SiC. The residual free silicon phase from this process causes microscopic hardness non-uniformity within the material, posing significant challenges for subsequent ultra-precision grinding and polishing [14,15]. On the other hand, the MEX technology demonstrates immense potential in SiC optical components due to its low equipment cost and the ability to manufacture highly customizable internal lattices [12,13]. Although limited by initial macroscopic surface defects such as the ’staircase effect’ caused by the nozzle extrusion process, as well as strict debinding and sintering shrinkage management, SiC mirrors fabricated via MEX have been successfully applied in substantive engineering stages, such as lasercom telescopes, through optimized post-processing.

### 2.2. Mirror Materials

#### 2.2.1. Traditional Mirror Materials

Space optical systems are subjected to severe mechanical vibrations and shocks during the launch phase, and must withstand extreme cyclic thermal environments during on-orbit operations. Consequently, mirror material selection must strike a delicate balance between specific stiffness and thermo-mechanical stability to minimize gravity-induced deformation and ensure the long-term stability of the surface figure accuracy [28]. Traditional space mirror materials are primarily categorized into two major systems: non-metallic materials, represented by SiC, glass-ceramics, and fused silica; and metallic materials, typified by aluminum and beryllium alloys [4].

Table 2 compares the key optomechanical and physical properties of conventional mirror materials. As indicated, in terms of mechanical properties, beryllium exhibits the highest specific stiffness, whereas aluminum exhibits the lowest. Regarding thermophysical properties, SiC provides a favorable combination of high thermal conductivity, low coefficients of thermal expansion (CTEs), and high specific stiffness, whereas fused silica combines a very low CTE with relatively low thermal conductivity. Glass-based materials, such as ULE and Zerodur, continue to dominate owing to their well-established fabrication processes, ultra-low CTE, and the capability to yield exceptionally high-quality optical surfaces. Concurrently, the fabrication and processing technologies for silicon carbide are maturing rapidly, establishing SiC as the most promising mirror material for space optical systems. Notably, with the maturation of single-point diamond turning (SPDT) technology, the application of metallic materials in the front-end components of catadioptric systems has become increasingly prevalent. Aluminum and beryllium–aluminum alloys not only possess excellent machinability but also enable the direct generation of high-precision optical surfaces via SPDT, thereby significantly facilitating monolithic optomechanical design [3,4].

**Figure 3:**
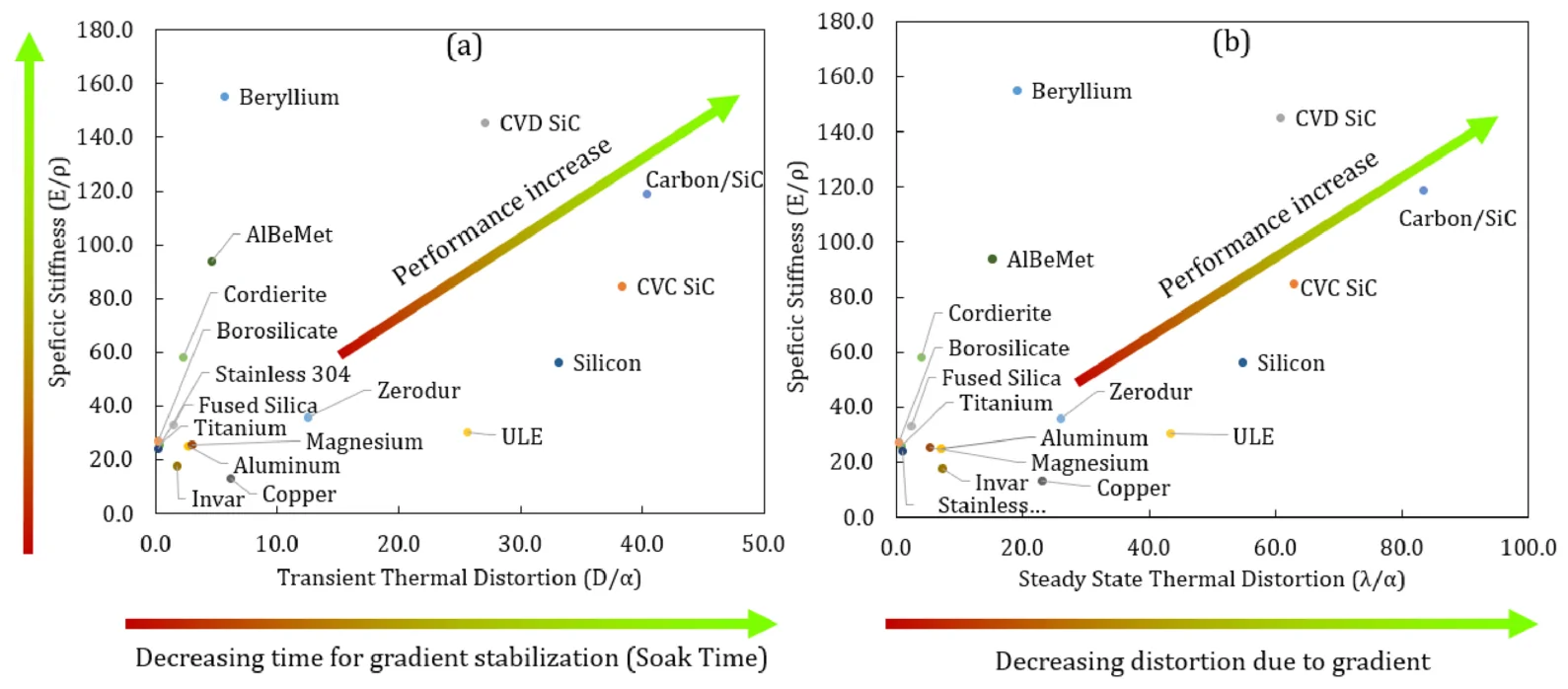
Figures of merit for common materials used in telescopes: (**a**) specific stiffness (E/ρ) versus diffusivity (*D*) per thermal expansion (α); (**b**) Specific stiffness (E/ρ) versus conductivity (λ) per thermal expansion (α). Reproduced with permission from [31] © Optical Society of America.

For an intuitive comparison, Horvath and Davies [31] evaluated these materials based on key metrics within a unified chart. Figure 3 illustrates the performance of various materials, plotting the specific stiffness (E/ρ) on the vertical axis against the transient and steady-state thermal dimensional stabilities on the horizontal axes. In Figure 3a, the horizontal axis represents D/α (where *D* is thermal diffusivity in ${\mathrm{mm}}^{2}/s$), with higher diffusivity and lower CTE corresponding to faster transient thermal gradient homogenization and lower instantaneous thermal strain. In Figure 3b, the horizontal axis denotes λ/α, where higher thermal conductivity combined with lower thermal expansion coefficient reduces steady-state thermal gradients and strain under given temperature differences.

As depicted in Figure 3, SiC exhibits favorable performance in both metrics compared with the other materials. After appropriate surface modification, SiC mirrors can achieve surface figure errors suitable for visible-light applications [32], elucidating why SiC ceramics are frequently adopted as substrate materials for optical mirrors. Recently, the Changchun Institute of Optics, Fine Mechanics and Physics (CIOMP) successfully developed the world’s largest monolithic SiC mirror blank, featuring an aperture of Φ4.03m [33], thereby achieving a monumental breakthrough in the fabrication technology for large-aperture RB-SiC mirror blanks. Nevertheless, the manufacturing of SiC mirrors remains prohibitively expensive, time-consuming, and technically demanding, making it difficult to meet rapid production requirements. Conversely, glass-based mirrors offer the advantages of excellent optical processability and superior surface figure accuracy; however, they are hindered by poor specific stiffness, high brittleness, and inherent challenges in weight reduction [34].

#### 2.2.2. AM Mirror Materials

AM mirror materials must not only satisfy the stringent requirements of conventional space mirrors for high specific stiffness and thermal stability, but also possess process-specific printability suitable for different AM routes, including powder flowability, laser absorptivity, slurry rheology, and photopolymerization response. Currently, AM mirror materials are broadly classified into two primary categories: metallic (e.g., AlSi10Mg) and non-metallic (e.g., SiC) materials.

From an optomechanical design perspective, the core advantage of AM materials lies in liberating designs from the geometric constraints imposed by conventional machining. By introducing lightweight architectures such as TO and lattice structures, material distribution and structural efficiency can be improved while reducing mass, thereby helping to maintain mirror surface–figure stability under gravitational or thermal loads. In addition, metallic AM materials are particularly well suited for monolithic mirror–support integration. Such integrated designs can reduce heterogeneous-material interfaces and the associated CTE mismatch, thereby improving system thermal stability and reducing assembly complexity. However, from a fabrication standpoint, achieving optical-grade surface accuracy necessitates that AM mirrors undergo complex post-processing. Metallic mirrors require a combination of SPDT, magnetorheological finishing (MRF), and surface modification coating processes to eliminate print-through textures and reduce surface roughness [35]. Conversely, SiC-based ceramics must undergo debinding and densification sintering processes. Table 3 compares the key performance parameters of typical AM mirror materials.

Because the properties of AM materials are strongly dependent on the manufacturing process, build orientation, and post-processing condition, Table 3 summarizes representative material properties together with the corresponding manufacturing and post-processing states. Where state-specific properties cannot be obtained from the original literature or material datasheets, they are indicated as “NR” rather than being replaced with generic bulk-material properties.

As indicated in Table 3, among metallic materials, aluminum alloys such as AlSi10Mg and AlSi12 exhibit low density and high specific stiffness, establishing them as the preferred candidates for AM metallic mirrors. Research indicates that SLM-fabricated AlSi10Mg exhibits high relative density and a fine-grained microstructure, resulting in mechanical properties superior to those of traditional cast aluminum alloys. Furthermore, it demonstrates near-isotropic behavior following appropriate heat treatment [41]. Consequently, this material holds immense potential in the realm of AM metallic mirror fabrication and is widely regarded as an ideal choice for manufacturing high-precision optical mirrors. Importantly, optimized SLM processing can enable AlSi10Mg components to achieve high relative densities, thereby reducing the prevalence of macroscopic porosity and improving the mechanical consistency and structural reliability of the mirror substrate. However, restricted by the inherent polycrystalline structure and the distribution of silicon phases within the aluminum alloy, direct polishing—even of highly dense substrates—still induces significant surface scattering due to differential wear. Therefore, an amorphous modification layer, such as electroless nickel–phosphorus (NiP), is typically necessitated to meet the rigorous optical surface requirements for visible wavelengths [42].

Among non-metallic materials, SiC has garnered extensive attention from researchers worldwide owing to its unique advantages of high specific stiffness and excellent thermal stability. Although AM techniques enable the fabrication of a wide array of complex SiC mirrors, current AM SiC components still encounter critical challenges regarding fracture toughness, which restricts their reliability under extreme launch environments [43]. To address this optomechanical engineering challenge, recent research efforts have been dedicated to incorporating reinforcement technologies—such as continuous fibers [44], chopped fibers/whiskers [45], and sandwich structure toughening [46]. These approaches aim to significantly enhance the structural integrity and damage tolerance of AM SiC mirrors without compromising their inherent optical stability.

In summary, while Table 3 illustrates the application potential of various AM technologies across multiple mirror materials, current research and substantive manufacturing are predominantly concentrated on aluminum alloys (specifically AlSi10Mg) and SiC due to constraints regarding optical performance requirements and process maturity. Consequently, the subsequent sections of this review will primarily focus on these two principal material systems.

### 2.3. AM Mirror Technology Roadmap

#### 2.3.1. Metal Mirrors: From Near-Net Forming to Optical Finishing

The AM process chain for metallic mirrors is a systematic engineering endeavor that integrates heat treatment and precision machining. A typical fabrication workflow encompasses critical stages including optomechanical design optimization, AM forming, stress relief and aging treatment, thermal cycling, SPDT, ultra-precision polishing, coating, and optical metrology [47]. Among these, the heat treatment stage serves as a crucial bridge connecting AM forming with optical cold working. It is of paramount importance for eliminating residual printing stresses and ensuring the stability of the surface figure following SPDT processing.

**Figure 4:**
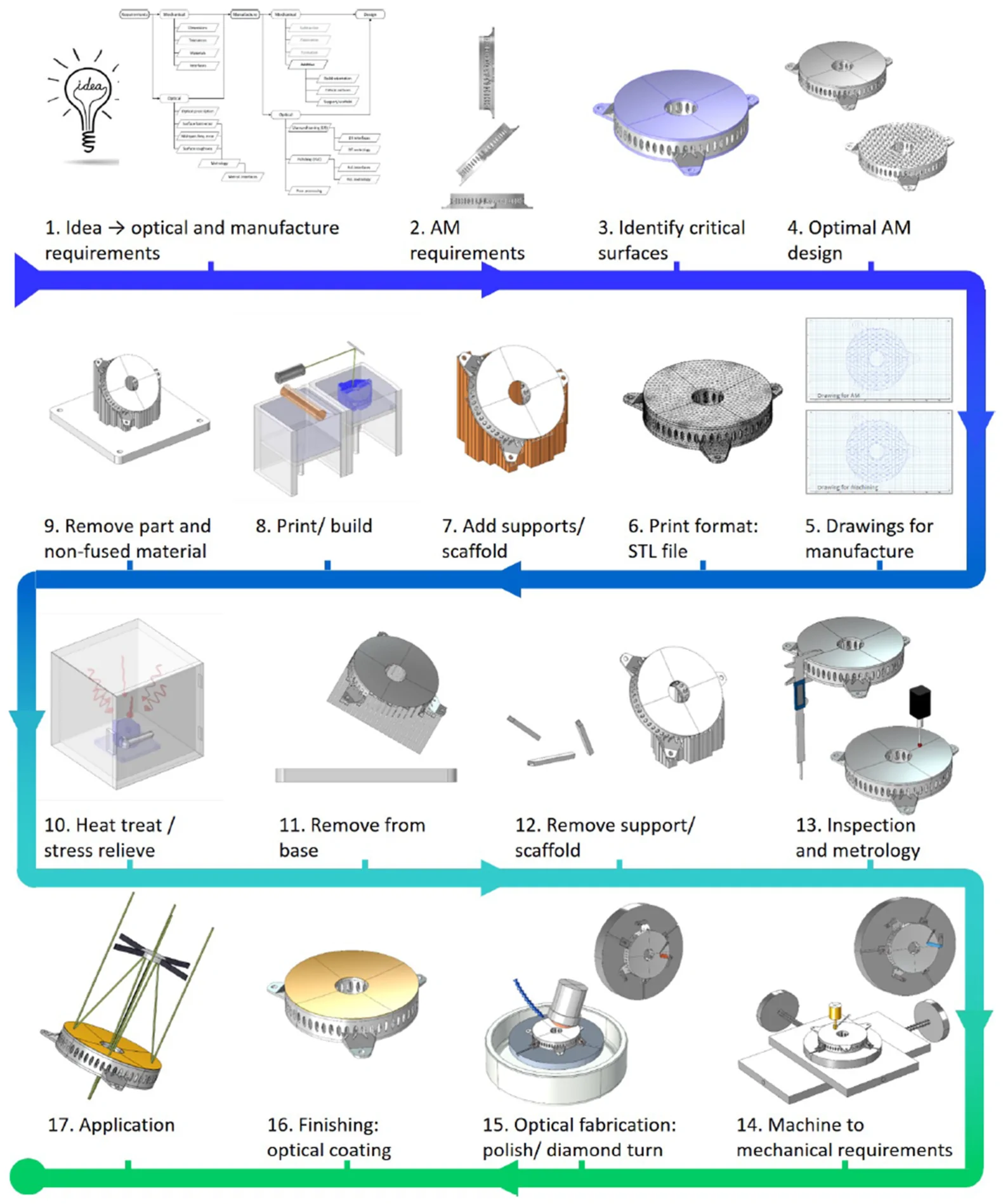
The design and manufacturing process chain for an AM metal mirror [49].

In practical optical applications, the manufacturing process chain must be custom-tailored according to the target operational wavelength bands. For instance, mid-wave and long-wave infrared (MWIR/LWIR) systems, owing to their longer operational wavelengths, exhibit a higher tolerance for surface roughness. Consequently, optical surfaces directly machined via SPDT (with $R_{a}\approx 5–10\;\mathrm{nm}$) typically suffice to meet the imaging quality requirements, obviating the need for an additional modification layer. Conversely, for visible and short-wave infrared (VIS/SWIR) applications, to satisfy the more stringent scattering and roughness criteria ($R_{a}<2\;\mathrm{nm}$), it is imperative to deposit NiP modification layer onto the substrate, followed by ultra-precision polishing to achieve a mirror-grade surface finish [48]. The detailed manufacturing process chain for metallic mirrors is illustrated in Figure 4 [49].

#### 2.3.2. SiC Mirrors: From Green Body to Dense Optical Component

**Figure 5:**
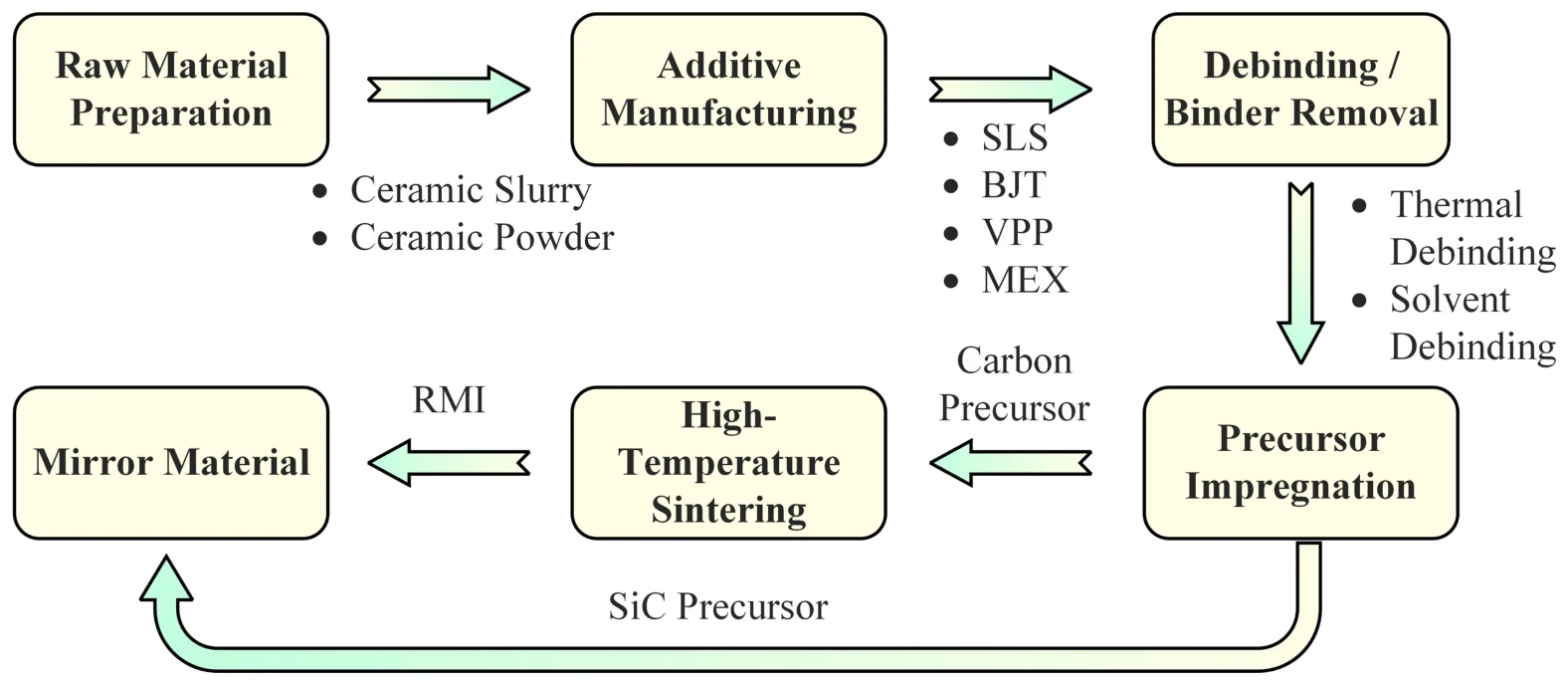
Technology roadmap for fabricating SiC mirrors based on AM technology.

For SiC mirrors with complex architectures, the integration of SLA and RMI technologies emerges as a highly effective approach for achieving high-precision manufacturing. The core of this process lies in synergizing the high forming resolution of SLA with the near-net-shape densification capability of RMI. Research by Li et al. [50] demonstrates that introducing a carbon-source precursor impregnation step after debinding can significantly elevate the SiC content within the sintered body. This effectively reduces the CTE and enhances thermal conductivity, which are paramount for the thermal stability of space mirrors. The typical technological roadmap for fabricating AM SiC mirrors is illustrated in Figure 5, which can be conceptualized into five stages from the green body to the dense optical component [20]:

Step 1 Raw Material Preparation: Typically involves slurry or powder feedstock;

Step 2 Additive Manufacturing: Selection of appropriate AM processes based on material form and required manufacturing precision to obtain green SiC mirror preforms;

Step 3 Debinding/Binder Removal: Organic binders are pyrolyzed into gases and carbon, resulting in porous SiC preforms;

Step 4 Precursor Impregnation: This stage employs either carbon precursors or SiC precursors. The former effectively increases the pyrolytic carbon content within the porous SiC preform; conversely, the latter, through multiple cycles of impregnation and pyrolysis of SiC precursors, achieves densification of the porous SiC mirror material and enhances its overall properties;

Step 5 High-Temperature Sintering: Utilizing RMI technology, liquid silicon infiltrates the porous skeleton and reacts with the carbon to synthesize dense SiC. This ultimately yields a highly densified SiC mirror blank possessing high specific stiffness and exceptional optical processability.

## 3. Research Status of AM for Space Mirrors

### 3.1. AM Mirrors Based on Conventional Lightweight Structures

**Figure 6:**
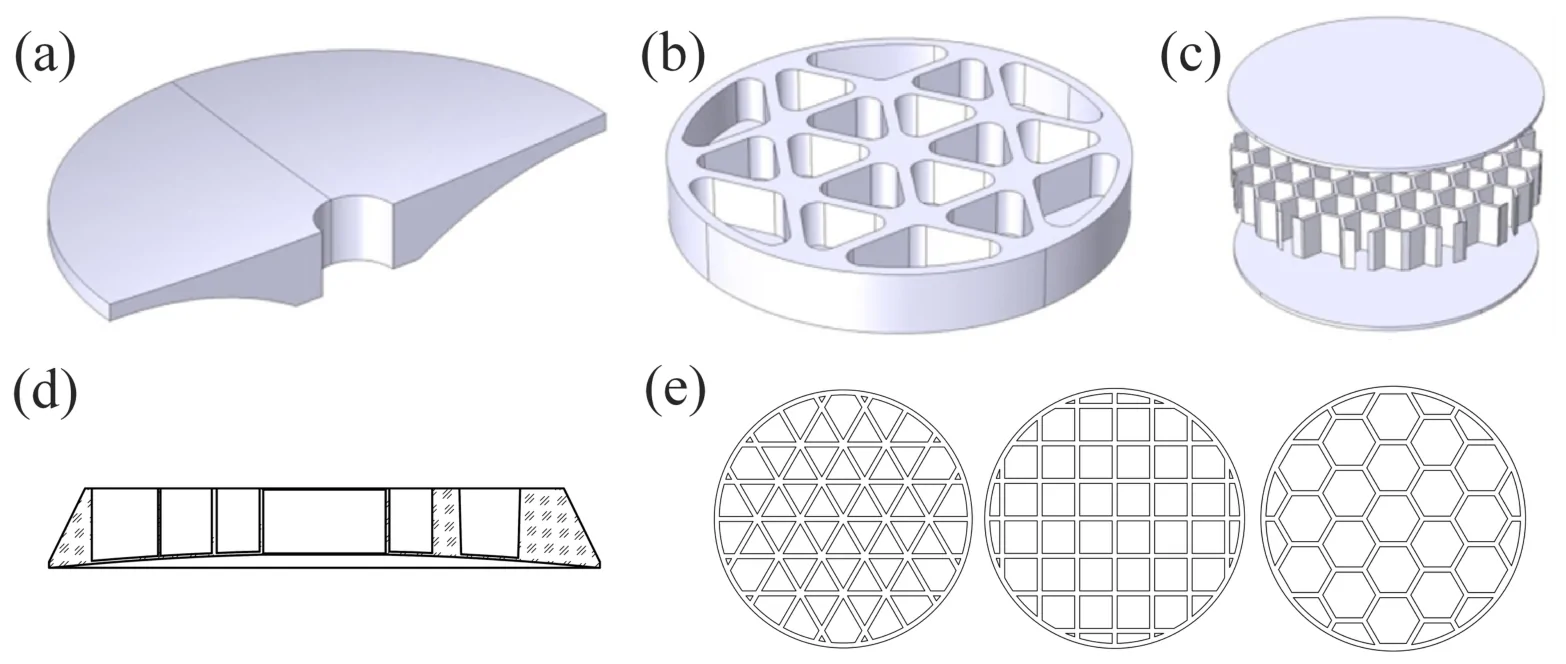
Conventional lightweight mirror designs: (**a**) an arched mirror, (**b**) an open-back mirror, and (**c**) a sandwich mirror [4]. (**d**) a cross-sectional schematic of an open-back mirror. (**e**) three reinforcing-rib configurations for lightweight mirrors.

Conventionally designed lightweight mirrors refer to design typologies manufacturable via traditional machining processes, which can be broadly classified into three categories: Arched, Open-back, and Sandwich configurations [51], as illustrated in Figure 6a–c. Among these, Arched mirrors achieve lightweighting by machining away a portion of the material from the mirror’s rear surface. While this structure is straightforward and easy to fabricate, it yields the lowest lightweight ratio; consequently, its application is becoming increasingly rare in practical engineering projects with stringent weight constraints. Open-back mirrors attain lightweighting through the machining of lightweight pockets on the rear surface. This configuration significantly enhances the degree of lightweighting, albeit at the expense of time-consuming and labor-intensive pocket machining. Compared to Open-back mirrors, Sandwich mirrors incorporate an additional planar support sheet, which improves the overall strength and stiffness of the mirror. However, this simultaneously exacerbates manufacturing complexity, making it challenging to control production costs and efficiency.

The lightweight pockets in Open-back and sandwich mirrors typically adopt triangular, hexagonal, or rectangular geometries, with their structures depicted in Figure 6d,e. Because lightweight mirrors with triangular configurations offer a higher lightweight ratio, whereas those with hexagonal configurations provide superior stiffness, these two designs are more prevalently utilized in practical engineering applications [52].

**Figure 7:**
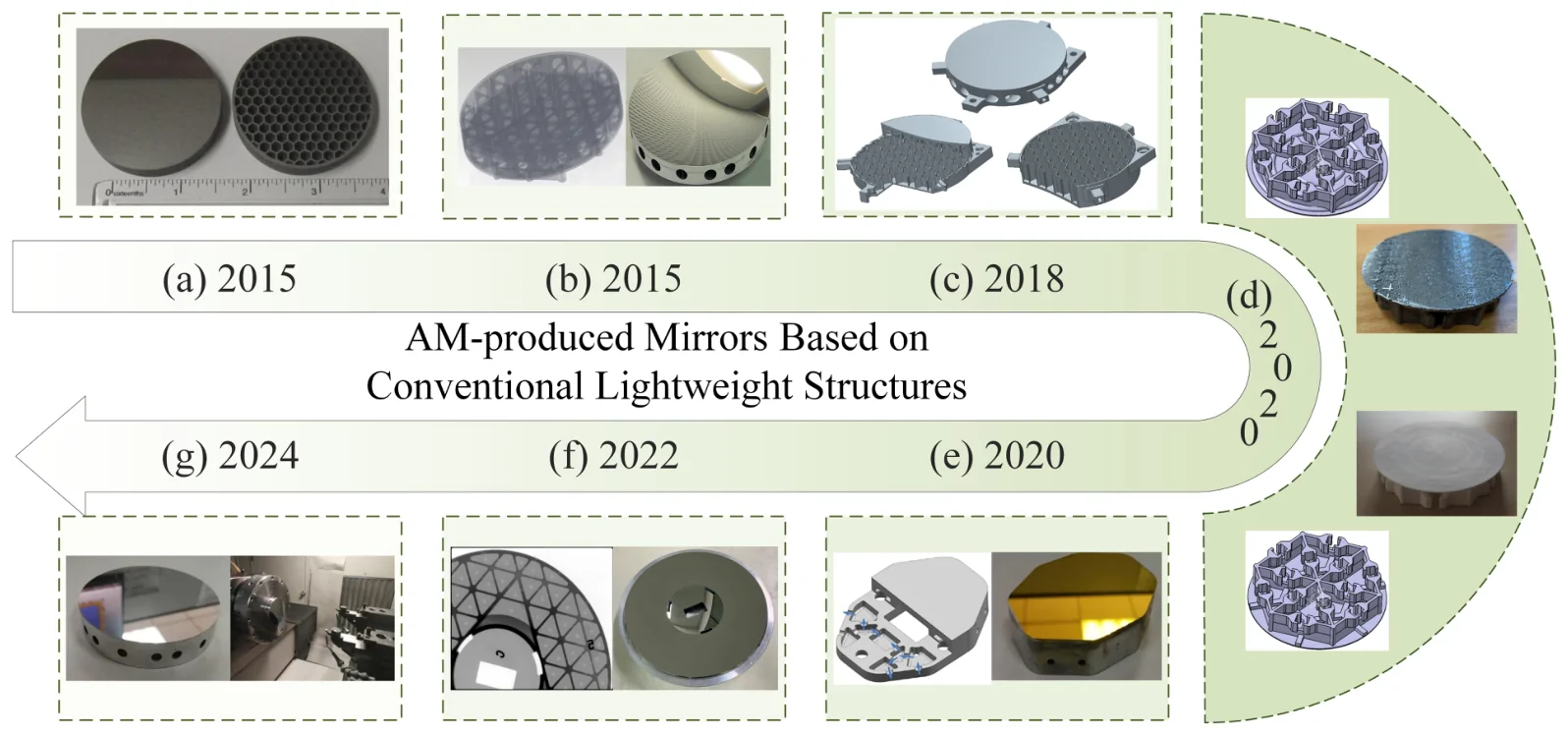
AM mirrors based on conventional lightweight structures: (**a**) honeycomb-structure mirror [53]; (**b**) model of open-back honeycomb mirror [54]; (**c**) model of sandwich honeycomb mirror [55]; (**d**) mirror with embedded actuators [24]; (**e**) infrared optical system mirror [47]; (**f**) integrated primary–tertiary mirror [56]; (**g**) high surface-density mirror [57].

In 2015, Corning Inc. [53] fabricated a high-performance aluminum mirror featuring a honeycomb lightweight structure using the direct metal laser sintering (DMLS) process. This approach significantly reduced the time required to machine lightweight pockets and improved manufacturing efficiency, as illustrated in Figure 7a. Through an analytical comparison of various mirror back architectures, Valente and Vukobratovich [51] discovered that sandwich mirrors exhibit greater stiffness than Open-back mirrors at an equivalent mass reduction level. In 2015, Sweeney et al. [54] proposed a lightweight design and AM methodology for sandwich mirrors. The interior of the mirror was filled with triangular ribs, and interconnected holes were incorporated at the center of the ribs to facilitate powder removal and further reduce mass, as shown in Figure 7b. In 2018, Hilpert et al. [55] from the Fraunhofer Institute designed and evaluated drilled mirrors, open-back honeycomb mirrors, and sandwich honeycomb mirrors. Their results indicated that the sandwich honeycomb mirror is superior in terms of lightweight ratio, stiffness, and stability, as depicted in Figure 7c. In 2020, Roulet et al. [24] from Aix Marseille Université investigated two embedded-actuator mirror concepts based on an open-back architecture. Ti-6Al-4V and alumina (Al_2_O_3_) prototypes were fabricated using EBM and SLA, respectively, as shown in Figure 7d. Numerical simulations of the optimized circular and honeycomb support configurations predicted polishing-induced surface form errors below 30 nm RMS under a polishing pressure of 3.5 MPa.

In 2020, Tan et al. [47] from CIOMP utilized SLM to fabricate a closed-back AlSi10Mg mirror for an infrared optical system. Measuring 67 mm × 50 mm, the mirror achieved a weight reduction of 43.7%. The measured RMS surface figure error after six months of storage was 58.85 nm, while the mean surface roughness after SPDT was $R_{a}$ = 7.244 nm, as shown in Figure 7e. In 2022, Zhang et al. [56] also proposed a composite closed-back mirror tailored for micro-satellites, which monolithically integrates the primary mirror (PM) and tertiary mirror (TM) of the optical system, thereby substantially saving space, manufacturing time, and launch costs. Concurrently, powder removal holes were introduced on the backplate to facilitate powder extraction. Following SPDT, surface modification, and polishing, the surface figure accuracies of the tertiary and primary mirrors reached measured values of 57 nm and 64 nm (RMS), respectively, as illustrated in Figure 7f. In 2024, Tan et al. [57] fabricated an AM mirror with high areal density utilizing a closed structure, as shown in Figure 7g. By employing methods such as polishing and coating, its applicability across different spectral bands was expanded, rendering this technology highly suitable for cryogenic detection applications.

### 3.2. AM Mirrors Based on TO Technology

**Figure 8:**
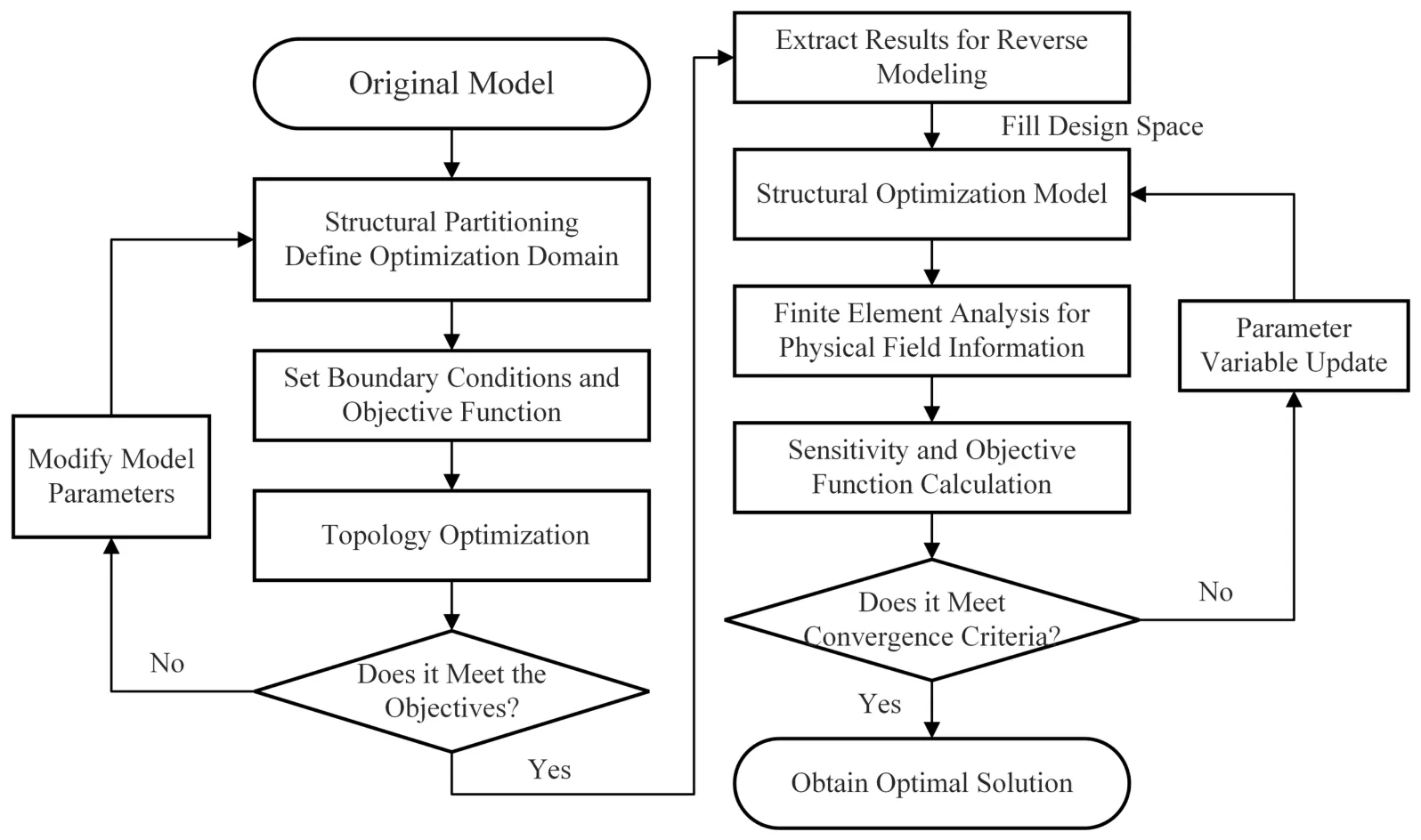
Topology Optimization design flowchart.

**Figure 9:**
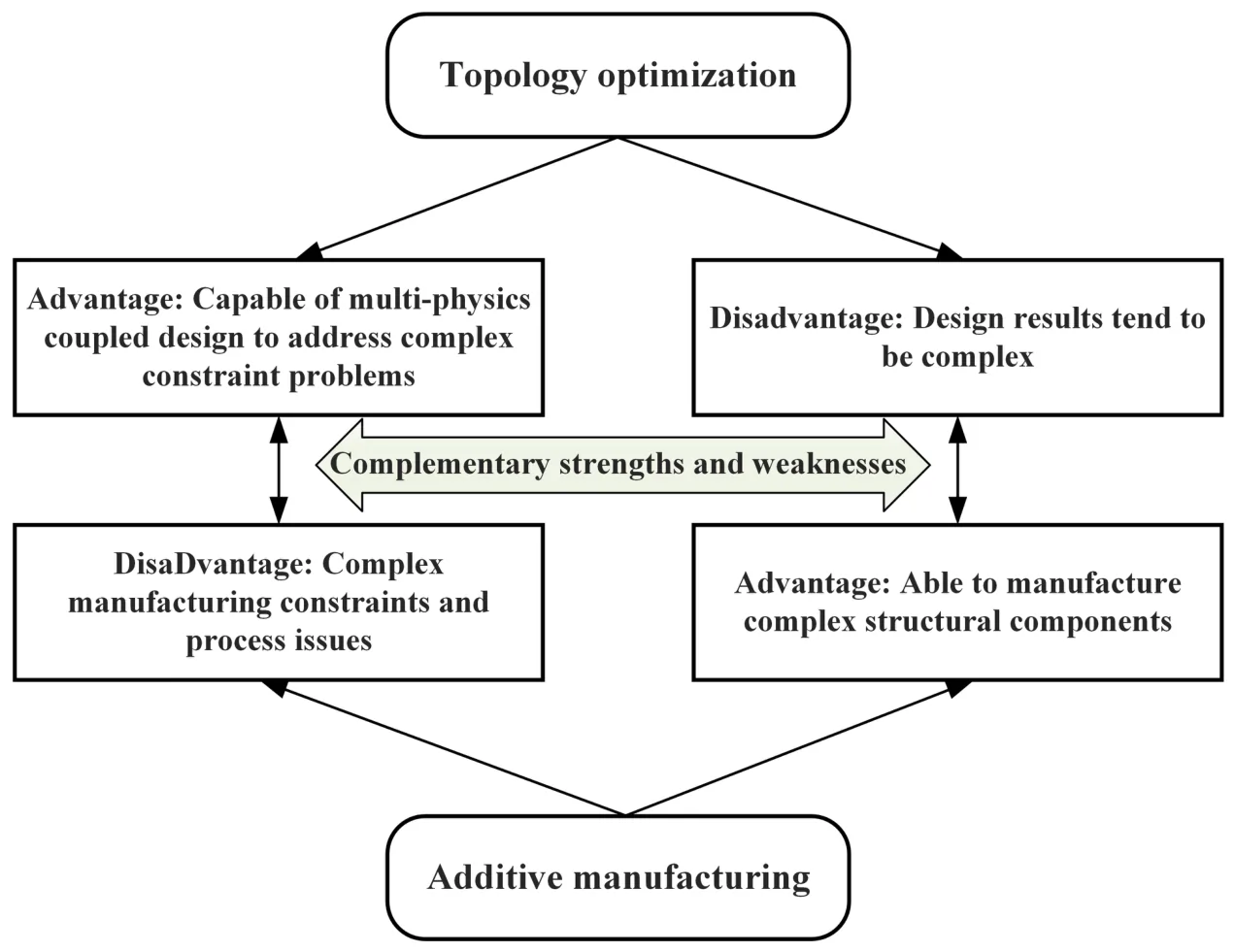
Characteristics of Topology Optimization and Additive Manufacturing.

TO is a structural optimization methodology employed to determine the optimal spatial distribution of material within a defined design domain. Subject to specific material properties, boundary constraints, and loading conditions, TO seeks the optimal material layout and the most efficient load-bearing paths to achieve high-performance lightweight designs [8]; its optimization design flowchart is illustrated in Figure 8. When applying the TO approach to the lightweight design of mirrors, the optimal geometric configuration is determined based on the specified design volume, loading conditions, and target stress criteria. This methodology enables the computational simulation and derivation of mirrors that surpass traditional designs in both structural stiffness and lightweight ratio. Furthermore, TO architectures can be thoroughly integrated with multi-physics coupled designs. However, the resulting optimized geometries are frequently highly complex. Leveraging AM processes effectively overcomes this manufacturability hurdle, creating a synergistic complementarity between the two technologies [58]. A comparison of their respective characteristics is also depicted in Figure 9.

**Figure 10:**
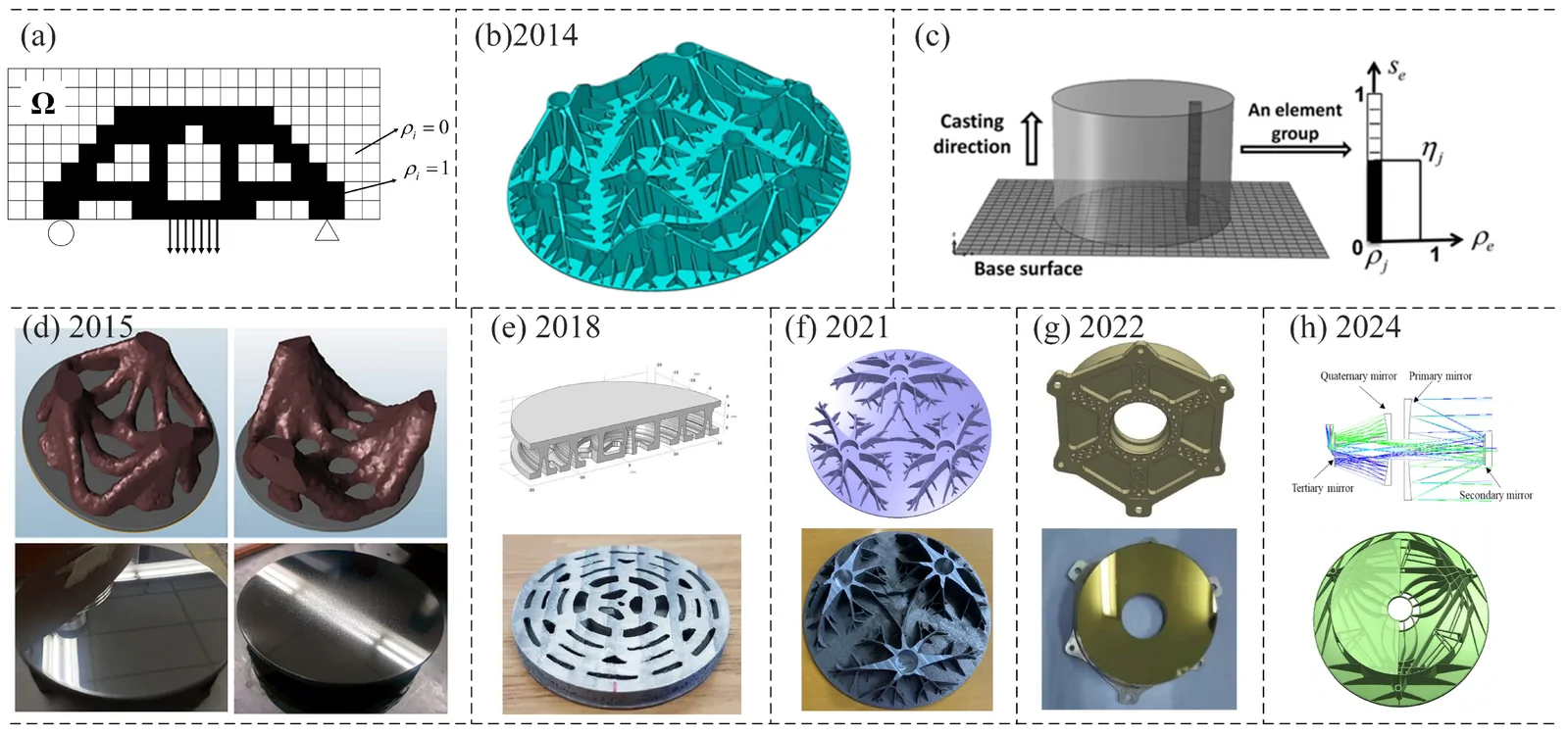
(**a**) TO schematic diagram. (**b**) 2 m large-aperture SiC mirror model, and (**c**) element relative density parameterization. Adapted with permission from [62] © Optical Society of America. (**d**) Top-mounted model with aluminum mirror surface and side-mounted model with titanium mirror surface [23]. (**e**) Coaxial tapered ring mirror [63]. (**f**) 260 mm topology-optimized mirror [64]. (**g**) Primary mirror assembly by AM [65]. (**h**) Coaxial four-mirror optical system and double-sided SiC mirror structure [66].

The density-based method is a prevalent approach in continuum TO. Its fundamental concept involves introducing a hypothetical material with variable density and designating this material density as the design variable, thereby transforming the structural TO problem into an optimal material distribution problem [59]. The Solid Isotropic Material with Penalization (SIMP) model is a widely adopted interpolation scheme within this method [60,61]. Grounded in the principles of continuum topology optimization, a relative density factor $\rho_{i}$ is assigned to each finite element, where $\rho_{i}$ takes a value of 1 or 0 to indicate the presence or absence of material within the element. Figure 10a illustrates the topological model of such a structure. Concurrently, a penalization factor *p* is introduced to penalize intermediate density values, driving them toward the binary extremes of 0 and 1. This mechanism facilitates a robust convergence from a continuous-variable TO model to a discrete optimization model. The elemental material properties (e.g., the elastic modulus) can be defined by Equation (1):

(1) $$E_{e}=\left(\underline{\rho }+\left(\bar{\rho }-\underline{\rho }\right)\rho_{e}^{p}\right)E_{0},\;e=1,2,\ldots ,N_{\mathrm{ele}}.$$

where $\underline{\rho }$ = 0.001 is a small lower bound value set to avoid computational singularity, $\bar{\rho }$ = 1 is the upper bound, $N_{\mathrm{ele}}$ is the total number of elements, $\rho_{e}$ and $E_{e}$ denote the relative density and elastic modulus of the *e*-th element, respectively, $E_{0}$ is the elastic modulus of the given isotropic solid material, and *p* is the penalty factor.

In addressing the TO of stiffening ribs on the rear of mirrors, beyond employing the binary variables (0 or 1) inherent to the SIMP method to delineate the spatial distribution of the ribs, it is imperative to parameterize their heights. Liu et al. [62] incorporated a parameterized Heaviside function into the TO process to characterize the rib height, successfully designing a novel SiC mirror featuring tree-like stiffening ribs and a bidirectional multi-arch backplate. The 2 m aperture mirror achieved a predicted RMS surface deformation of 12.55 nm in the finite element analysis (FEA), as depicted in Figure 10b. The parameterization principle for the elemental relative density utilizing the Heaviside function is illustrated in Figure 10c, and the corresponding mathematical model can be formulated as Equation (2):

(2) $$\begin{array}{c} \rho_{e}=\rho_{j}\times H\left(s_{e},\eta_{j}\right),\\ \mathrm{where}\;H\left(s_{e},\eta_{j}\right)=\left\{\begin{array}{cc} 1, & s_{e}<\eta_{j},\\ 0, & s_{e}\geq \eta_{j}, \end{array}\right.\;j=1,2,\ldots ,N_{\mathrm{eg}}. \end{array}$$

where $\rho_{j}$ is the design variable, taking a value of 1 or 0 to indicate whether the *j*-th element group exists or not; *H* is the Heaviside function; $\eta_{j}$ is the design variable that defines the position of the solid–void interface within the *j*-th element group; $s_{e}$ is the normalized coordinate of the element center; $N_{\mathrm{eg}}$ is the number of element groups in the design domain.

In 2015, Herzog et al. [23] from the University of Arizona conducted topology optimization for lightweight design, adopting minimized surface displacement as the optimization objective. They designed two mirror configurations featuring top- and side-mounting mounts. Aluminum and titanium alloy mirror blanks were subsequently fabricated utilizing DMLS and EBM processes, respectively, followed by fine grinding and polishing of the optical surfaces, as shown in Figure 10d. In 2018, Atkins et al. [63] from the UK Astronomy Technology Centre designed a topology-optimized arched lightweight mirror utilizing FEA tools. The resulting optimized internal infill comprises a series of concentric conical rings radiating outward from the center; its structural model is depicted in Figure 10e.

In 2021, Fan et al. [64] accomplished the TO design of a 260 mm aperture primary mirror for spaceborne remote sensing cameras. Fabricated via SLM technology and subsequently processed by SPDT, the mirror achieved a measured RMS surface figure error of 25.94 nm; its structural configuration is illustrated in Figure 10f. In 2022, Yan et al. [65] synergized AM with TO to realize the monolithic design and fabrication of an AlSi10Mg primary mirror assembly, which encompassed both the mirror substrate and the backplate. This approach effectively resolved the degradation of optical surface accuracy and stability induced by disparities in material parameters—such as mounting stresses and thermal mismatch—thereby achieving passive integration and an athermal design for the mirror assembly. The finalized assembly weighed merely 36.3 g. Simulation analysis indicated that, under gravity-induced loading, the mirror exhibited a predicted RMS surface deformation below 1 nm. When a mounting-surface flatness of 0.003 mm was imposed, the predicted RMS surface deformation remained below 5 nm. The corresponding structure is depicted in Figure 10g. In 2024, Shen et al. [66] employed TO to design a double-sided SiC mirror tailored for a coaxial four-mirror system, as presented in Figure 10h. Featuring a facesheet thickness of 2.5 mm, the design accommodated a PM with a 510 mm clear aperture and a quaternary mirror (QM) with a 300 mm clear aperture, adhering to a strict total mass constraint of 5.18 kg. Notably, this innovative architecture effectively mitigates the print-through effect typically induced by the non-uniform rib distributions characteristic of TO.

### 3.3. AM Mirrors Based on Lattice Structures

**Figure 11:**
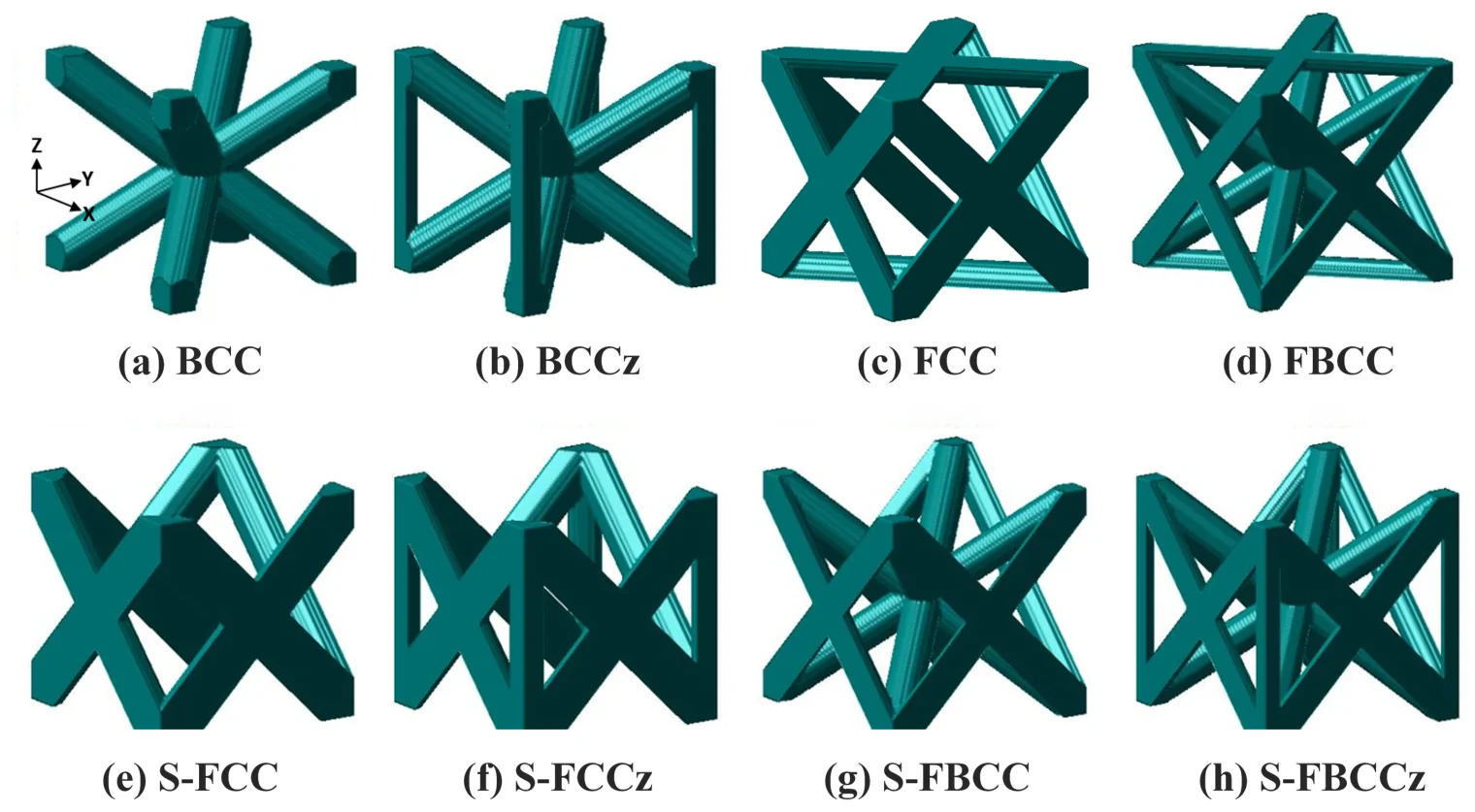
Library of truss-based unit cells. BCC is body-centered cubic; BCCz is BCC with *z* direction reinforcement; FCC is face-centered cubic; FBCC results from the union of FCC and BCC; names with ‘S’ prefix are self-supporting variants which have no members that lie parallel to the *x*–*y* plane [68].

A lattice structure typically consists of intersecting struts or strips that delineate square or diamond-shaped interstitial voids [67], representing one of the earliest and most extensively adopted configurations in parameterized design. Common lattice topologies include body-centered cubic (BCC) and face-centered cubic (FCC) unit cells, among others, as illustrated in Figure 11 [68]. Ultralight lattice architectures can achieve a lightweight ratio exceeding 85%, boasting exceptional specific stiffness, specific strength, and superior energy absorption capabilities [69]. Consequently, they have found widespread application across the aerospace, biomedical, and electronics sectors.

In the structural analysis of strut-based designs, the Maxwell number can be used as a geometric indicator to distinguish between stretch-dominated and bending-dominated behavior [70]. For an ideal three-dimensional pin-jointed framework, the Maxwell number *M* is given by Equation (3):

(3) M=b−3j+6

where *b* and *j* denote the numbers of struts and joints, respectively. Under this idealization, M≥0 generally indicates a stretch-dominated topology, whereas M<0 indicates a tendency toward bending-dominated behavior [71,72]. For an ideal two-dimensional pin-jointed framework, the corresponding Maxwell criterion is given by Equation (4):

(4) M=b−2j+3

For 2.5-D extruded architectures, the applicability of the 2D criterion depends on whether their load-bearing topology can reasonably be idealized as a planar pin-jointed framework. Moreover, for rigidly connected or monolithically fabricated lattices, where the joints transmit bending moments, the deformation mode cannot be determined solely from the Maxwell criterion and should instead be evaluated using beam/frame mechanics or numerical analysis [72].

**Figure 12:**
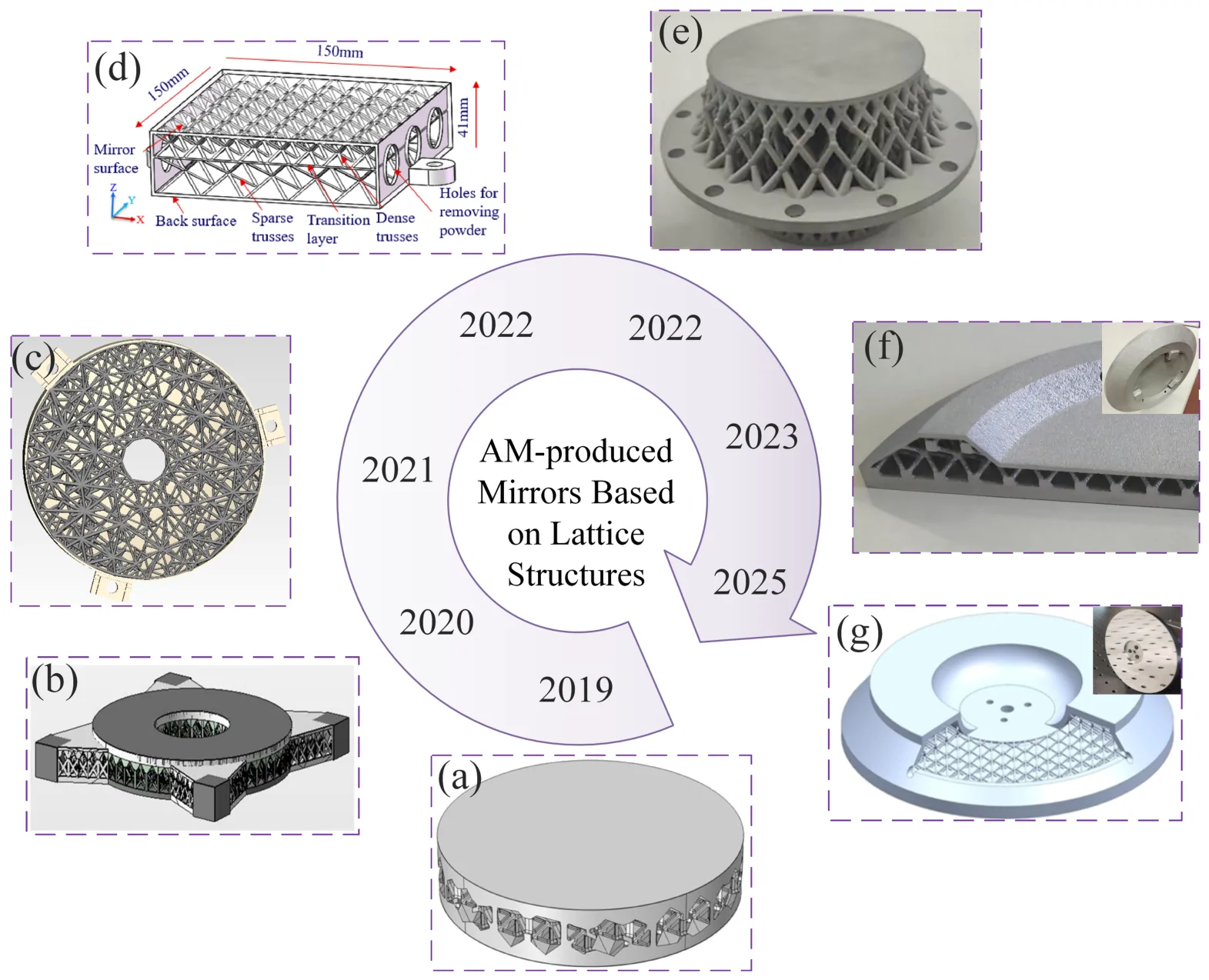
(**a**) The mirror structure comprising the hexagonal cells [73]. (**b**) Complete parametric view of the mirror [74]. (**c**) Non-uniform lattice mirror after optimization [75]. (**d**) Cut view of the mirror model with double-layer truss structure [76]. (**e**) SDG lattice infilled mirror [77]. (**f**) Sectioned mirror [78]. (**g**) Cross-sectional view of a 150 mm aperture mirror [79].

To achieve mirrors with superior specific stiffness, numerous scholars have adopted approaches that synergize lattice structures with topology optimization [68]. In 2019, Atkins et al. [73] integrated lattice structures with TO to create a mirror configuration lighter than uniform lattices. This structure discretized the mirror into an assembly of honeycomb-like unit cells and achieved lightweighting by subtracting the scaled volumes of rhombic dodecahedrons from the lattice structure. This resulted in a lightweight ratio of 49.1% and a predicted RMS surface deformation of 0.3 nm in the FEA, as shown in Figure 12a. The following year, Snell et al. [74] built upon this foundation to propose a novel annular lattice structure based on a cylindrical coordinate system. Applied to an 72 mm diameter mirror, this approach achieved a mass reduction of 37%, as depicted in Figure 12b.

In 2021, Zhang et al. [75] from the Beijing Institute of Space Mechanics & Electricity applied lattice structures to the structural design of small-aperture aluminum alloy mirrors. Taking the cross-sectional area of the lattice struts as the optimization objective, they employed the moving iso-surface threshold (MIST) TO method to optimize the relative density distribution of the lattice. The optimized mirror surpassed traditional lightweight configurations in both lightweight ratio and surface figure accuracy; its structure is shown in Figure 12c. In 2022, Liu et al. [76] designed and fabricated a square closed-back metallic mirror featuring a double-layer truss structure. Measuring 150 mm × 150 mm and equipped with powder removal holes on its sidewalls, the mirror achieved a remarkable lightweight ratio of 85%, with its structure illustrated in Figure 12d. Following post-processing of the printed mirror blank, its measured RMS surface figure error was 17.72 nm. That same year, Yang et al. [77] from the Beijing Institute of Technology (BIT) utilized a density-based TO method to design and manufacture a double-sided mirror with a size and diameter gradient (SDG) lattice structure. This design conserved 20% of the material while yielding superior stiffness and anti-vibration performance, as depicted in Figure 12e.

In 2023, Boschetto et al. [78] from Sapienza University of Rome utilized SLM technology to fabricate a closed-back metallic mirror with a lattice infill structure, featuring micro-holes on its rear surface for internal powder removal, as shown in Figure 12f. However, this structure exhibited a relatively low lightweight ratio, reducing the mass by only 25% compared to a solid counterpart. In 2025, Tan et al. [79] analyzed the characteristics of modification materials and proposed a pure aluminum modification layer scheme tailored for cyclic thermal environments, successfully fabricating a 150 mm diameter aspheric AlSi10Mg mirror. Incorporating a BCC lattice lightweight infill structure, the mirror achieved a measured RMS surface figure error of approximately 12 nm following surface modification and optical cold working, as illustrated in Figure 12g. It maintained excellent surface figure stability even after thermal cycling tests. This research provides critical technical guidance for achieving superior surface structures in the processing of additively manufactured mirror blanks.

In summary, although lattice structures have been extensively utilized in mirror designs owing to their exceptionally high lightweight ratios and design flexibility, their inherent structural limitations warrant significant attention. Conventional lattice unit cells are predominantly configured from simple strut- or truss-based topologies, rendering it challenging to precisely manipulate such complex structures through a limited set of global macroscopic parameters. Furthermore, the intersections of these strut-based architectures are highly susceptible to localized stress concentrations. Most importantly, traditional variable-density lattice structures typically exhibit discrete, step-wise density distributions, which often fall short of meeting the stringent requirements for smooth, continuously graded infill structures in high-end optical mirrors. These technical bottlenecks impede the further enhancement of the comprehensive performance of lattice-based mirrors, necessitating further in-depth exploration by researchers.

### 3.4. AM Mirrors Based on TPMS Technology

**Figure 13:**
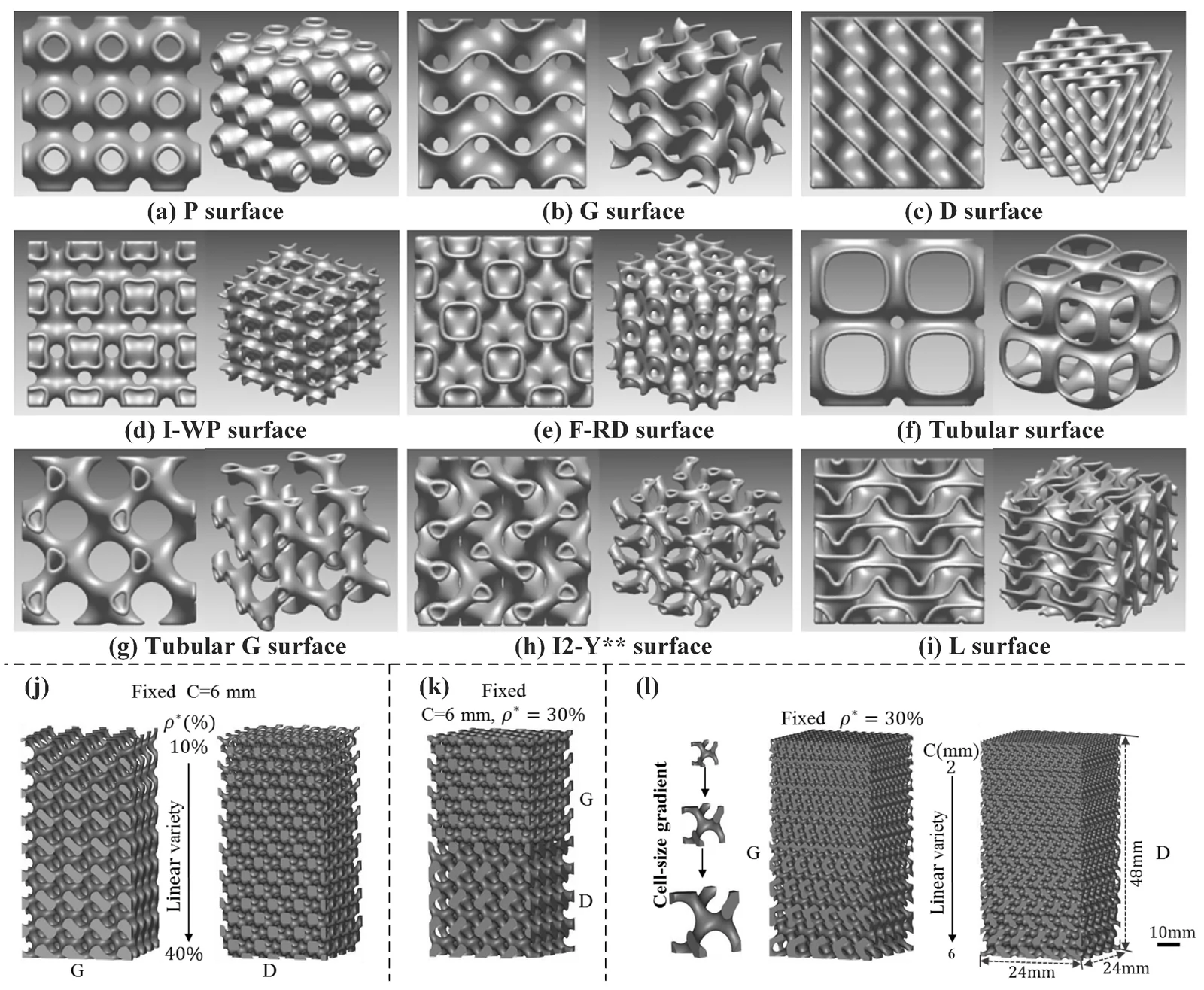
TPMS structures: (**a–i**) nine types of TPMS structures expressed by different implicit functions [80]. (**j**) Gradient samples in relative density, (**k**) gradient samples in heterostructure and (**l**) gradient samples in cell size [84]. The double asterisk ** in I2-Y** is part of the surface designation used in the original reference and does not denote a footnote or statistical significance. The superscript asterisk * in ρ* denotes relative density and is part of the variable notation, rather than a footnote or statistical significance symbol.

Triply periodic minimal surfaces (TPMS) are smooth, fully interconnected, periodic porous shell-like structures formulated via implicit functions, characterized by a mean curvature of zero at all points, as illustrated in Figure 13a–i [70,80]. Owing to their geometric smoothness, TPMS unit cells significantly mitigate stress concentrations, exhibiting exceptional mechanical properties, superior shock absorption and energy dissipation capabilities, controllable structural parameters, and high specific surface areas. Furthermore, TPMS lattice architectures possess inherent self-supporting characteristics, obviating the need for additional support structures during the AM process. This not only enhances production efficiency but also provides a novel design paradigm for the complex lightweighting of optical mirrors [81,82,83].

The performance enhancements afforded by conventional uniform TPMS structures to mirrors are somewhat limited. Considering the spatial variations in internal stress and stiffness within mirrors, numerous researchers have begun exploring functionally graded TPMS lattice structures, most notably through integration with TO methods. Liu et al. [84] provided the first systematic description of the variation in unit cell size gradients along specific paths. Based on network-type TPMS architectures, they proposed three gradient modes: density gradient, heterogeneous structure gradient, and cell size gradient, as depicted in Figure 13j–l. Zhou [85] from Chongqing University comprehensively detailed the mechanical properties of graded lattice structures synergizing TPMS with the SIMP algorithm. They discovered that, at an equivalent mass, the stiffness of the shell-like graded lattice infill structure is markedly superior to that of conventional periodic lattice structures.

**Figure 14:**
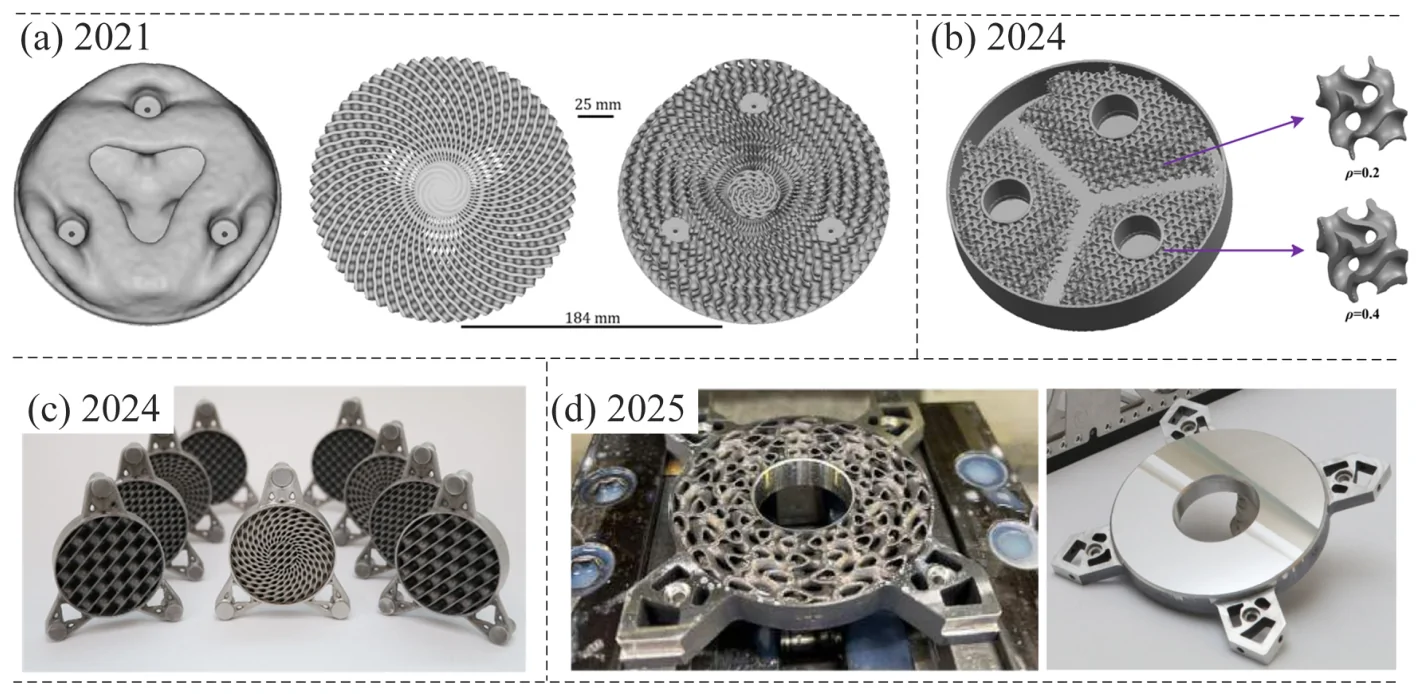
(**a**) A mirror filled with a sheet-diamond TPMS lattice. Reproduced with permission from [31] © Optical Society of America. (**b**) A 400 mm-aperture TPMS-filled reflective mirror [86]. (**c**) A 52 mm-aperture mirror incorporating a TPMS structure [87]. (**d**) CCam M1 structure integrated with TPMS [88].

In 2021, Horvath and Davies [31] from Oak Ridge National Laboratory synergized TO, lattice infill, and analytical estimation methods. They discovered that sheet-based diamond TPMS lattice structures exhibit excellent stiffness, subsequently applying them to the design of AM lightweight mirrors, as shown in Figure 14a. The mirror, fabricated from SiC, featured a facesheet thickness of 1 mm and a total mass of 0.45 kg. FEA of the 184 mm SiC mirror predicted an RMS self-weight surface deflection of 0.9 nm. This provides a novel paradigm for future mirror fabrication and design. In 2024, Zeng et al. [86] from the University of Electronic Science and Technology of China (UESTC) proposed a TPMS structural optimization design method based on a homogenization performance model. This approach can accurately describe the structure and achieve exceptional performance at minimal computational cost. Utilizing a structural control method based on a wall-thickness field, they designed a TPMS-filled SiC mirror with a diameter of 400 mm and a height of 60 mm, achieving a mass reduction of approximately 70%, as shown in Figure 14b. FEA indicated that the predicted RMS surface deformation was below 20 nm at room temperature and remains below 30 nm RMS at 50 °C. During the comparison with a traditional open-back mirror of equivalent mass, the authors found that the designed TPMS-filled mirror exhibited superior performance when the temperature reached 50 °C. This is attributed to the flexible structure of the TPMS, which better absorbs thermal deformation caused by temperature fluctuations.

In 2024, Lister et al. [87] from the UK Astronomy Technology Centre (UK ATC) conducted the design of aluminum alloy mirrors incorporating TPMS architectures. The mirror, with a diameter of 52 mm and a depth of 10 mm, utilized an internal thin-walled lattice design. Two design variants achieved lightweight ratios of 50% and 70% for the prototypes, respectively, with the mirror structures depicted in Figure 14c. In 2025, Aziz et al. [88] outlined the design, manufacturing, and evaluation processes for AM aluminum alloy mirrors, selecting the PM (M1) of the CubeSat Camera (CCam)—developed by RAL Space and UK ATC—as the design subject. This mirror features an annular structure with an outer diameter of 84 mm and an inner diameter of 32 mm, along with a clear aperture of 80 mm. The optimized infill structure employed a TPMS Split-P cylindrical unit cell lattice mapping architecture. The physical mirror was printed via the L-PBF process using AlSi10Mg. The resulting mirror achieved a lightweight ratio of 56% and good optical surface quality with surface roughness <8nm RMS, as illustrated in Figure 14d. Notably, during the post-processing of the mirror, it was discovered that while hot isostatic pressing (HIP) could reduce surface porosity, it simultaneously increased surface roughness, thereby exacerbating optical scattering—a critical issue that warrants further investigation and resolution.

Evidently, compared to conventional strut-based lattice structures—which are highly susceptible to nodal stress concentrations and exhibit discrete, step-wise density distributions—TPMS offer a highly promising, continuously graded lightweighting solution for space mirrors, owing to their smooth, fully interconnected, and zero-mean-curvature geometric topologies. TPMS not only fundamentally mitigates localized stress concentrations, demonstrating exceptional specific stiffness, inherent self-supporting printability, and robust thermal stability; but more crucially, when deeply synergized with TO, it enables smooth gradient distributions. This effectively circumvents the print-through effect frequently induced by conventional macroscopic stiffening ribs.

However, the highly complex and tortuous internal micro-channels inherent to TPMS significantly exacerbate the difficulty of extracting un-melted powder. Furthermore, although densification post-processing can reduce the porosity of the mirror blank, it is highly prone to degrading the substrate’s surface roughness and exacerbating optical scattering. Consequently, achieving a global synergy between the micro-parameterized design of TPMS and subsequent post-processing techniques—such as stress-relief annealing and SPDT—represents a vital research trajectory for the future fabrication of high-performance AM space mirrors.

### 3.5. AM Mirrors Based on Voronoi Architectures

**Figure 15:**
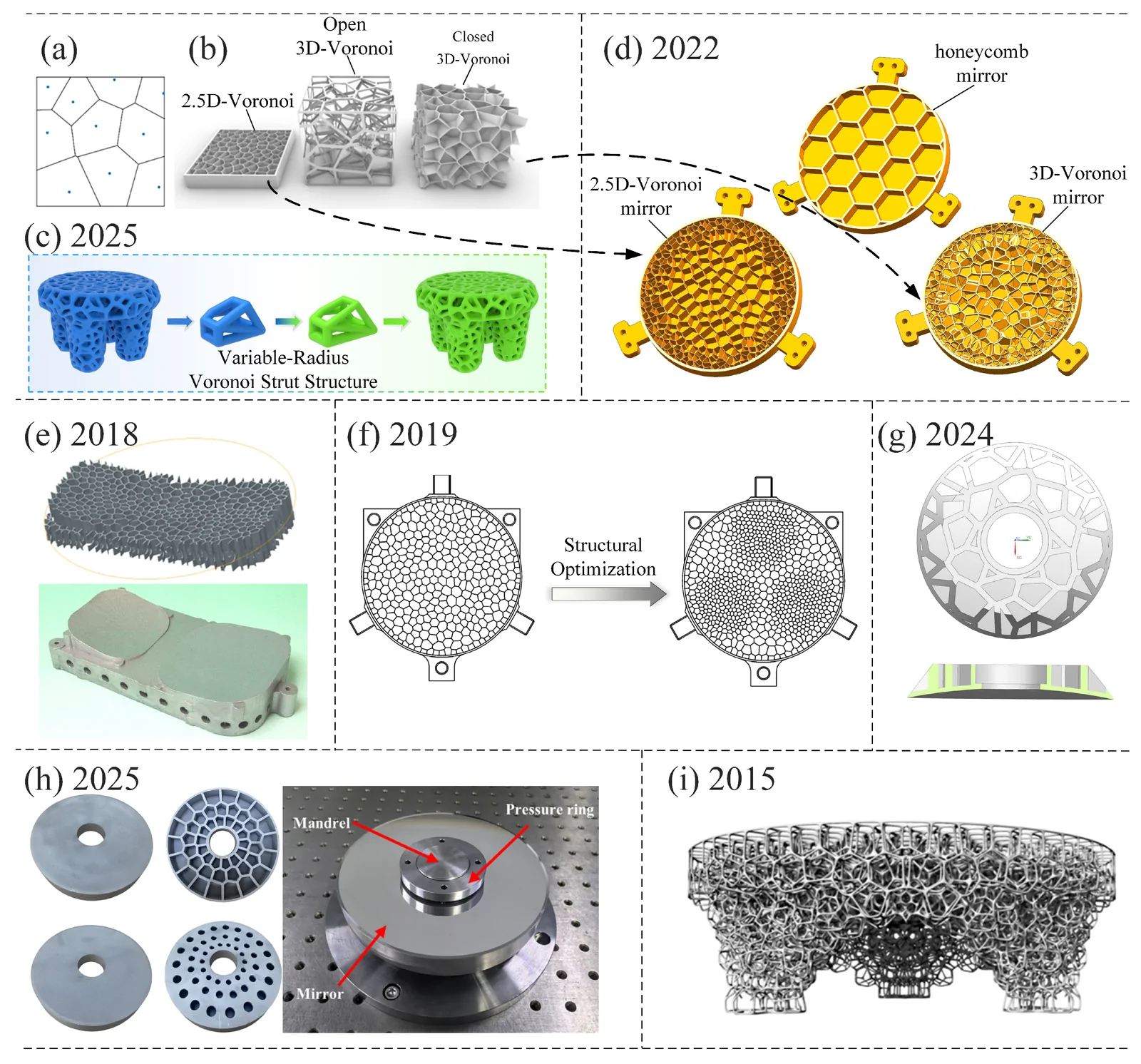
Representative Voronoi-based lightweight mirror designs: (**a**) Schematic diagram of Voronoi structure generation and (**b**) from left to right: 2.5D-Voronoi, open 3D-Voronoi and closed 3D-Voronoi [91]. (**c**) Stress-driven variable-radius Voronoi strut structure [92]. (**d**) From left to right: honeycomb mirror, 2.5D-Voronoi mirror and closed 3D-Voronoi mirror [91]. (**e**) Mirror with compound surfaces [93]. (**f**) Optimization process and demonstrator mirror for a 76 mm-aperture mirror [42]. (**g**) 288 mm-aperture 2.5D-Voronoi metal mirror. Adapted with permission from [94] © Optica Publishing Group. (**h**) 150 mm-aperture 2.5D-Voronoi metal mirror. Adapted with permission from [95] © Optica Publishing Group. (**i**) Voronoi-based structure with 1000 points generated using Lloyd’s algorithm [96].

The Voronoi diagram, formulated by mathematician G. F. Voronoi in 1905, comprises a continuous polygonal tessellation delineated by the perpendicular bisectors of line segments connecting adjacent points. A fundamental mathematical property of this structure is that any point within a given polygon is strictly closer to its corresponding generating control point (often referred to as a seed) than to any other control point within the domain, as illustrated in Figure 15a [89]. Utilizing Voronoi algorithms enables the generation of highly controllable 2D and 3D porous biomimetic architectures. These structures afford exceptional degrees of freedom in design optimization, exhibiting superior specific strength and robust energy absorption characteristics. Furthermore, they facilitate the realization of ultra-lightweight, fully interconnected frameworks that adeptly satisfy the stringent mass reduction constraints and performance metrics of optical mirrors. Consequently, such architectures have found extensive application across diverse sectors, including aerospace, biomedical engineering, and the automotive industry [90].

For a given seed points set in a space ${\left\{\Omega_{i}\right\}}_{i=1}^{n}$, the Voronoi tessellation of Ω can be defined as follows:

(5) $$V_{i}=\left\{x\in \Omega |∥x-p_{i}∥\leq ∥x-p_{j}∥,\forall j\neq i\right\}$$

where $V_{i}$ denotes the *i*-th Voronoi cell, *x* denotes an arbitrary point in the domain Ω, and $∥•∥$ represents the distance metric. Various forms of Voronoi structural units can be generated by modifying parameters such as the seed point set and the distance metric.

Synergizing Voronoi structural design with TO represents a pivotal approach in current optical mirror design. Topology-optimized Voronoi architectures can effectively minimize the unsupported areas of optical surfaces, thereby suppressing residual thermal stress deformations during the AM process and achieving superior lightweight designs. Based on spatial dimensionality, Voronoi mirrors are primarily categorized into 2.5D and 3D configurations. Furthermore, depending on the topological connectivity of their internal pores, 3D-Voronoi structures can be subdivided into open-cell and closed-cell architectures, as illustrated in Figure 15b,c.

In 2022, advancing beyond the limitations of conventional honeycomb lightweight configurations, Zhang et al. [91] integrated TO methods to design 2.5D-Voronoi and closed-cell 3D-Voronoi mirrors, as depicted in Figure 15d. FEA results demonstrated that, compared to conventional honeycomb configurations, these mirrors exhibited superior optomechanical performance: first-order modal frequencies increased by 20% and 25%, while load-induced surface deformations decreased by 23% (19.02 nm RMS) and 34% (16.90 nm RMS), respectively. This clearly highlights the Voronoi architecture’s advantages in enhancing structural stiffness and deformation resistance. However, the closed-cell 3D-Voronoi design traps un-melted powder within enclosed cavities, presenting a critical powder-removal challenge that must be addressed in future AM applications.

The 2.5D-Voronoi mirror configuration can be conceptualized as having internal stiffening ribs generated via the extrusion of a 2D-Voronoi tessellation. While geometrically three-dimensional, the rib architecture remains uniform, exhibiting no structural variation along the optical axis. At present, Voronoi-based mirrors are predominantly characterized by these 2.5D-Voronoi infill architectures. Notable representative works include the innovative integrated primary tertiary mirror design proposed by Heidler et al. [93] in Fraunhofer Institute for Applied Optics and Precision Engineering (IOF) for an off axis three-mirror-anastigmat (TMA) telescope, and the lightweight Voronoi filling structure for mirrors generated by Hilpert et al. [42] in 2019, as shown in Figure 15e,f. Both paradigms adopted a sandwich-structured design, strategically incorporating powder removal holes on the internal ribs and outer sidewalls. This approach maximizes the structural stiffness while rigorously ensuring the manufacturability of the additive process.

In 2024, Zhang et al. [94] proposed a modeling and optimization methodology for 2.5D-Voronoi porous structures, accomplishing the design of a metallic mirror with a 288 mm aperture. This mirror achieved a lightweight ratio of 71.12% and a predicted RMS surface deformation of 2.82 nm in the FEA, as shown in Figure 15g. In 2025, Chen et al. [95] from the Xi’an Institute of Optics and Precision Mechanics (XIOPM) proposed a biomimetic TO design method for high-specific-stiffness aluminum alloy mirrors based on Voronoi unit cell architectures. Featuring a 150 mm aperture, the mirror blank was successfully fabricated utilizing SLM technology, as illustrated in Figure 15h.

Currently, there are relatively few designs of mirrors based on open 3D Voronoi structures. In 2015, Mici et al. [96] generated an open 3D Voronoi structure with 1000 seed points inside a mirror using Lloyd’s algorithm and performed TO, as shown in Figure 15i. The structure can be optimized by adjusting the number of seed points and the dimensions of the structural members. Compared to closed structures, open 3D Voronoi designs allow the removal of residual powder from AM, and their porous cell configurations offer significant design freedom. However, due to their increased complexity and susceptibility to localized stress concentrations, these structures pose greater challenges for both design and fabrication. As a result, open 3D Voronoi mirrors remain largely in the theoretical research stage, yet represent an important direction for future development.

In 2025, Liu et al. [92] from Huaqiao University proposed a stress-driven dual-level generation method for constructing three-dimensional variable-radius Voronoi strut structures, as illustrated in Figure 15c. This approach utilizes implicit surface modeling to avoid geometric discontinuities caused by explicit Boolean operations. By coordinating macro global and cell local controls, it achieves precise regulation of relative density distribution and ensures smooth transitions between variable radius struts, effectively addressing issues such as strut connectivity and stress concentration.

Evidently, owing to their exceptional biomimetic porosity and immense topological degrees of freedom, Voronoi architectures are garnering widespread attention in lightweight space mirror design, driving a diversification of structural configurations. Nevertheless, current Voronoi-based designs still confront significant theoretical and engineering bottlenecks. Primarily, existing studies remain predominantly confined to 2.5D extruded configurations, underutilizing the ultimate potential of 3D spatial topologies; comprehensive research applying 3D-Voronoi strut-based and closed-cell structures to mirror design remains underexplored. Furthermore, the critical challenge of powder evacuation in closed-cell architectures must be resolved, potentially by incorporating interconnecting venting holes within cavity walls or adopting MEX processes. Concurrently, the deep integration of artificial intelligence (AI) and machine learning (ML) algorithms into the generation and parameter optimization of Voronoi unit cells—enabling intelligent generative designs driven by coupled optomechanical–thermal multi-physics—constitutes a crucial research frontier for the future design of biomimetic Voronoi mirrors.

### 3.6. Summary of Mirror Performance Fabricated by AM

In summary, based on lightweight topological types and internal infill architectures, the preceding sections have reviewed the recent research progress of AM mirrors. To intuitively compare the key technical specifications of various AM mirrors, this section selects representative research achievements since 2020 and categorizes them into simulation data and physical test data, presented in Table 4 and Table 5, respectively.

Table 4 summarizes the technical specifications of the FEA models for AM mirrors. The evolving data trends indicate that the apertures of mirrors at the design stage exhibit significant diversification and a tendency toward larger dimensions, spanning from 120 mm to 510 mm. This demonstrates that the feasibility of advanced topological structural design for large-aperture space mirrors is being thoroughly validated through high-precision finite element simulations. Regarding material systems and structural schemes, the simulation studies encompass two mainstream categories: ceramic-based (SiC) and metal-based (AlSi10Mg). Specifically, due to its exceptionally high specific stiffness and excellent thermal stability, SiC is frequently utilized for the lightweight reduction and optomechanical coupled topology optimization of large-aperture mirrors. Conversely, relying on their outstanding manufacturing flexibility, metallic materials such as AlSi10Mg demonstrate unique advantages in integrated multi-mirror assemblies or monolithic mirror-support structural schemes.

Compared to the theoretical designs, Table 5 comprehensively presents the engineering application outcomes of recent AM mirrors in terms of physical fabrication and testing. Regarding the breadth of manufacturing processes and materials, the fabrication of physical mirrors is not solely dependent on AlSi10Mg metallic materials but has successfully expanded to SiC ceramics, alumina ceramics, and novel modified aluminum alloys. The manufacturing capability for physical apertures has achieved a breakthrough, advancing from 50 mm miniature engineering prototypes to full-scale large-aperture SiC mirror substrates at the 500 mm level. This fully highlights the practical advancement of AM technology in manufacturing space optomechanical components. To address the core bottleneck of high initial surface roughness induced by the printing process, physical studies exhibit diversified surface modification strategies. In addition to the widely applied electroless NiP plating, Au coatings and modified Al layers have been actively explored, successfully realizing high-precision optical surfaces after fine polishing. Simultaneously, the lightweighting ratio of the fabricated mirrors is remarkably outstanding, reaching up to 85%. These results demonstrate the capability of AM to realize complex internal lightweight architectures while maintaining competitive structural and optical performance.

Table 4 and Table 5 summarize numerical predictions and experimentally measured performance, respectively. It should be emphasized that these two datasets are not directly comparable because they involve different mirror geometries, aperture sizes, boundary conditions, loading cases, manufacturing routes, and metrology conditions. Nevertheless, their juxtaposition qualitatively illustrates that excellent numerically predicted structural performance does not necessarily translate directly into the final optical surface quality of fabricated mirrors. In practice, the latter is additionally influenced by process-induced defects, residual stress, machining, surface modification, polishing, and measurement conditions.

## 4. Post-Processing Technologies

Due to the inherent layer-by-layer fabrication characteristics of AM, as-printed mirrors typically exhibit elevated surface roughness and internal defects, failing to meet the stringent surface quality requirements of optical remote sensing systems. Consequently, to actualize the critical transition from a macroscopic printed blank to a precision optical component, establishing a comprehensive post-processing workflow—proceeding through densification, stress relief, precision machining, and surface modification coating—is of paramount importance. This section will briefly outline the primary defect typologies and their corresponding post-processing methodologies.

### 4.1. Densification and Heat Treatment

**Figure 16:**
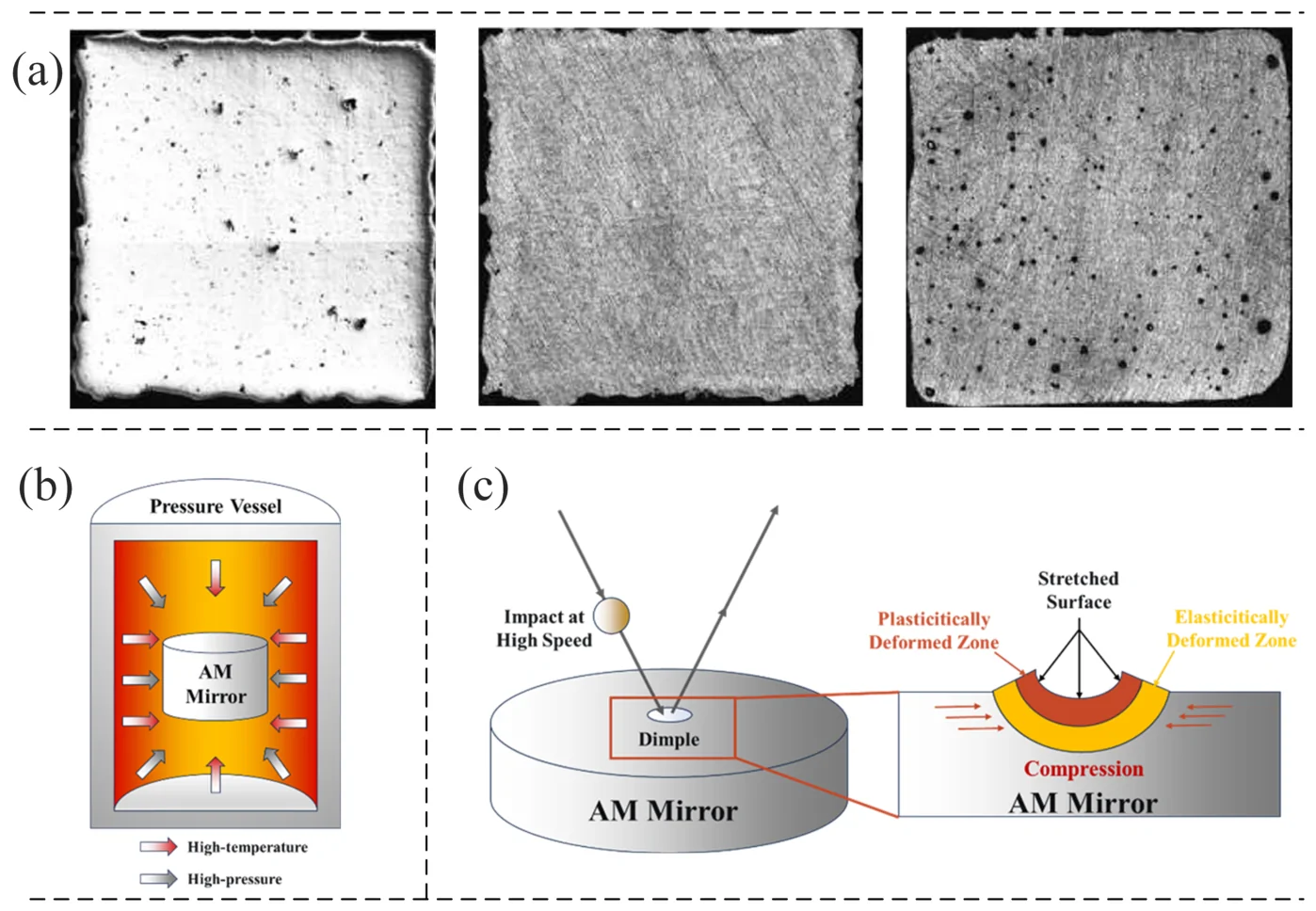
(**a**) Example cubes from the DoE showing LoF (**left**), low porosity (**middle**) and KH (**right**) [97]. (**b**) Schematic diagram of HIP. (**c**) Schematic diagram of mechanical peening.

Internal defects constitute a critical determinant affecting the structural stability of AM mirrors. In metallic mirrors, these internal anomalies predominantly encompass lack of fusion (LoF), keyholes (KHs), and gas-entrapped bubbles (GBs), as illustrated in Figure 16a. Conversely, for ceramic mirrors such as SiC, internal defects not only include micro-cracks and porosity but also residual unreacted Si and carbon particles. Porosity typically manifests as spherical cavities, whereas the propagation of micro-cracks is intricately associated with the thermophysical properties of the constituent materials [97]. During subsequent SPDT, these subsurface defects are exposed as surface pits, thereby inducing severe optical scattering on the mirror face. Consequently, implementing densification treatments prior to ultra-precision machining is of paramount importance.

HIP is an important densification process that can substantially reduce internal porosity and improve material density through the combined application of elevated temperature and isostatic pressure [24]. It constitutes a pivotal technology for achieving optical-grade mirror surfaces, with its operational principle illustrated in Figure 16b. Research indicates that as-built specimens devoid of HIP treatment exhibit conspicuous porosity defects following SPDT, whereas HIP-treated specimens achieve improved surface densification. However, HIP promotes the formation of silicon precipitates within the metal mirrors, resulting in increased surface roughness, which necessitates further subsequent polishing to resolve [88,98,99,100]. Notably, the HIP process necessitates the meticulous selection of treatment parameters; excessive pressure can compromise the dimensional accuracy of the workpiece, whereas inadequate pressure fails to achieve the requisite densification. Mechanical peening serves as an alternative post-processing methodology that induces severe plastic deformation on the material surface via mechanical impact to enhance densification, as depicted in Figure 16c. During the fabrication of metallic mirrors utilizing cold spray techniques, the deposited materials frequently suffer from high porosity, diminished interfacial bonding strength, and pervasive residual tensile stresses. To circumvent these issues, Maharjan et al. [101] employed two distinct mechanical peening processes—deep cold rolling (DCR) and controlled hammer peening (CHP)—to fortify cold-sprayed Ti-6Al-4V coatings deposited on a titanium alloy substrate. Their findings revealed that both peening modalities effectively augmented the coating-substrate bonding strength while concurrently achieving a profound 71% reduction in porosity.

For ceramic mirrors such as SiC, RMI is predominantly required to accomplish structural densification. RMI is a near-net-shape fabrication process that rapidly densifies ceramic matrix composites by infiltrating molten Si or silicon alloys into a carbonaceous preform, subsequently triggering an in-situ reaction to yield SiC. To further elevate the structural density, supplementary procedures, such as the impregnation of carbon-source precursors, are necessitated to maximize the final SiC yield.

Concurrently, the advent of advanced metrology and inspection technologies has significantly bolstered the fabrication quality of AM mirrors. Non-destructive testing (NDT) techniques, epitomized by X-ray computed tomography (XCT), have emerged as the gold standard for evaluating internal defects within AM components [88]. This technology enables the precise identification of the spatial distribution and volumetric characteristics of internal porosities, thereby providing robust empirical data to support the comprehensive evaluation and optimization of densification processes.

Even after achieving material densification, the residual thermal stresses induced by the extremely high cooling rates inherent to the AM process remain a primary barrier limiting the surface figure accuracy of the mirrors. If subsequent precision machining is executed directly without prior stress relief, the gradual relaxation of these residual stresses will cause the mirror’s surface figure to evolve dynamically over time or under varying temperatures, thereby severely degrading the optical imaging quality.

Thermal treatment represents an effective and widely adopted countermeasure for eradicating residual stresses, primarily encompassing conventional heat treatments (e.g., annealing, aging, and T6 treatment), cryogenic treatments, and thermal cycling treatments [102]. Among these, while conventional heat treatments demonstrate remarkable efficacy in stress relief, prolonged exposure to elevated temperatures can induce grain coarsening and alter the metallographic microstructure, thereby compromising the mechanical properties. In contrast, cryogenic treatment optimally reshapes the internal stress distribution while effectively preserving the hardness, strength, and microstructural stability of the material. Furthermore, thermal cycling induces alternating thermal stresses during repeated heating and cooling, thereby promoting the progressive redistribution and relaxation of residual stresses and stabilizing the material. Consequently, meticulously selecting an appropriate thermal treatment strategy is imperative for the effective stress relief of AM mirrors.

### 4.2. SPDT and Ultra-Precision Polishing

SPDT is a core precision forming process for AM metal mirrors, which can efficiently eliminate macroscopic defects such as the ’staircase effect’ on the as-printed surface and achieve a high-precision initial surface figure. However, the SPDT process inevitably leaves behind periodic tool marks [103]. These high-frequency spatial errors induce a grating effect that leads to severe optical scattering, which must be rigorously eliminated through subsequent ultra-precision polishing procedures. It should be noted that the ultra-precision polishing techniques and the corresponding numerical data discussed in this section serve as general examples, which are broadly applicable to both AM and standard conventional mirrors.

**Figure 17:**
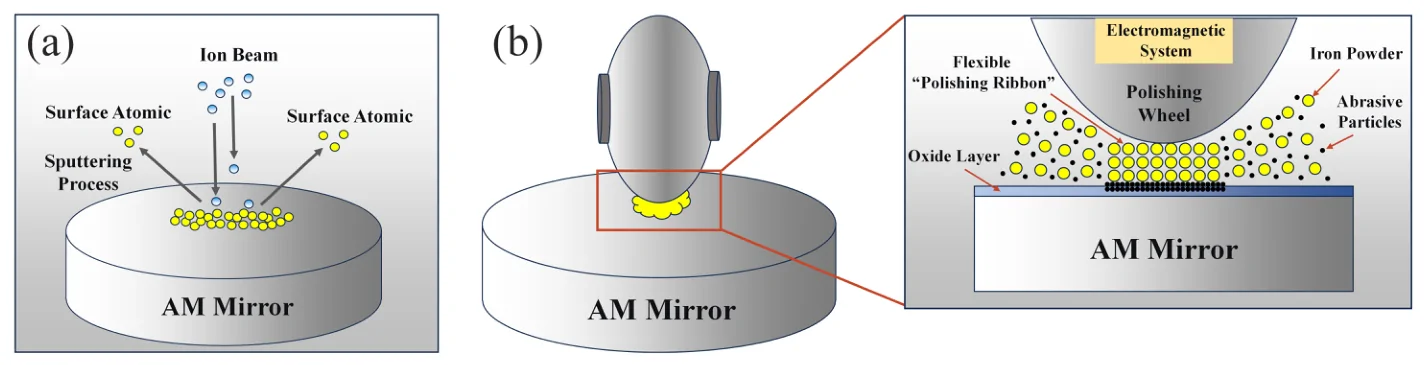
(**a**) Schematic diagram of IBF. (**b**) MRF removal mechanism.

To eradicate residual SPDT tool marks and further correct the surface figure, researchers have introduced a multitude of advanced polishing methodologies, such as chemical mechanical polishing (CMP), stress lap polishing (SLP), airbag polishing (ABP), ion beam finishing (IBF), and magnetorheological finishing (MRF) [104]. Among these, IBF and MRF stand out as highly prominent technologies for the high-precision polishing of optical mirrors. IBF is a non-contact machining technique that utilizes the sputtering effect of a high-energy ion beam to achieve high-precision, highly deterministic surface figuring of optical components. It boasts significant advantages, including the absence of mechanical contact, highly deterministic material removal, and the complete avoidance of polishing impurity introduction, as illustrated in Figure 17a. Conversely, MRF employs a magnetorheological fluid that instantaneously solidifies under an applied magnetic field to form a flexible polishing tool. It removes material primarily through hydrodynamic shear forces exerted under extremely low normal pressures. This technology enables the fabrication of ultra-smooth surfaces while introducing virtually no subsurface damage (SSD). It comprehensively combines the merits of high polishing efficiency, exceptional surface quality, and robust equipment and technological versatility, with its operational principle depicted in Figure 17b.

**Figure 18:**
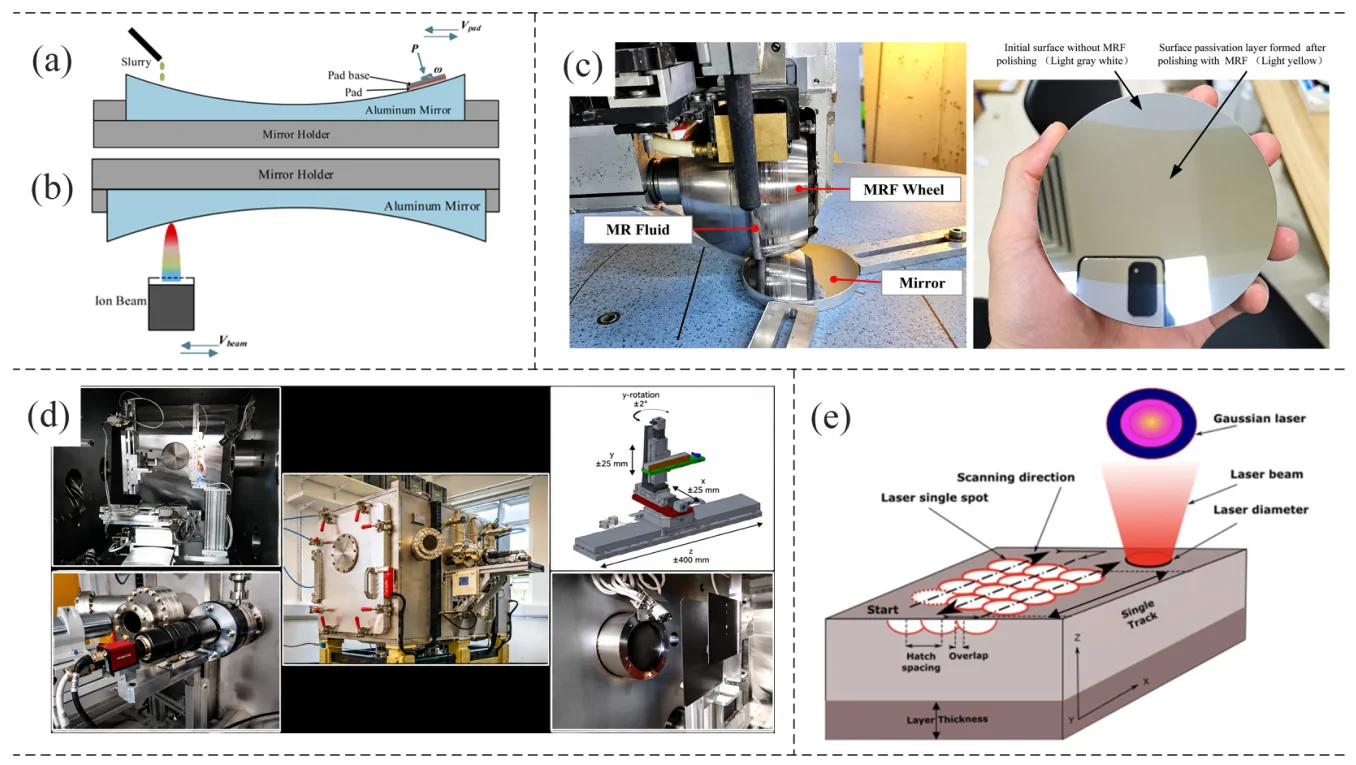
(**a**) Schematic diagram of the SP process and (**b**) Schematic diagram of the IBS process [103]. (**c**) MRF and CMP processing equipment [105]. (**d**) Photographs of the IBF system and its components: external view of the IBFsystem (center), internal view of the IBF system (upper left), schematic 3D model showing the motionstages, sample holder, and Faraday cup detector (upper right), high-resolution camera for sample alignment (lower left), and primary and secondary aperture plate mounts (lower right) [106]. (**e**) Schematic of the laser polishing process and its parameters [107].

Beyond the aforementioned mainstream polishing technologies, numerous scholars have actively explored a variety of hybrid polishing processes and novel polishing methodologies. Du et al. [103] proposed a hybrid process combining ion beam sputtering (IBS) and smoothing polishing (SP) to eradicate residual SPDT tool marks. Yielding a material removal efficiency significantly superior to that of direct polishing, this approach ultimately achieved an ultra-smooth, tool-mark-free surface with a measured roughness of 3.7 nm, as depicted in Figure 18a,b. Bai et al. [105] proposed a combined polishing strategy of MRF coupled with chemical mechanical polishing (CMP) tailored for aluminum alloy mirrors, alongside the development of a novel acidic nano-silica polishing slurry. Consequently, the measured surface figure error of the aluminum mirror was reduced from 63.28 nm to 15.19 nm, while the surface roughness ($R_{a}$) decreased from 3.6 nm to 1.38 nm, as shown in Figure 18c. Pradhan et al. [106] presented a non-contact deterministic polishing technique—IBF—with its system and components illustrated in Figure 18d. Ultimately, the measured surface figure irregularity of this NiP mirror was reduced to 1.9 nm RMS over an effective clear aperture of 20 mm × 10 mm.

Furthermore, laser polishing has garnered considerable attention as a novel technology for improving surface finish and enhancing component performance. This technique utilizes a laser beam to remelt the mirror surface, forming a smoother profile under the synergistic effects of gravity and surface tension. Nevertheless, this process imposes extremely stringent requirements on processing parameters such as laser power, scanning speed, and the number of remelting cycles, with its operational principle illustrated in Figure 18e [107].

### 4.3. Surface Modification Technologies

For substrate materials like aluminum alloys and SiC, achieving sub-nanometer optical surface roughness directly on the bare substrate is highly challenging due to inherent micro-porosity or multi-phase microstructures. Therefore, surface modification techniques are commonly employed to obtain superior surface quality. By depositing a dense coating of a new material onto the mirror substrate, this technique fundamentally improves the surface physical characteristics and conceals the inherent micro-porosity of the matrix. This lays a crucial foundation for subsequent optical precision machining, ensuring the ultimate realization of nanometer-level surface roughness. The predominant surface modification methods for optical mirrors primarily include chemical vapor deposition (CVD) and physical vapor deposition (PVD). CVD utilizes gaseous precursors that undergo chemical reactions on the substrate surface to synthesize a dense coating; while it offers uniform composition and controllable thickness, the reaction process is prone to generating hazardous byproducts. Conversely, PVD deposits thin films via physical vaporization mechanisms within a vacuum environment. It boasts significant advantages, such as a clean process and robust coating adhesion, although it imposes extremely stringent requirements on environmental cleanliness. Both are recognized as pivotal technologies for high-performance surface modification.

An ideal optical surface modification material typically requires the following critical attributes: (1) excellent machinability, facilitating the attainment of a nanometer-level mirror finish through optical fabrication; (2) a CTE that closely matches that of the substrate to avert thermally induced surface figure distortion, alongside robust environmental adaptability; and (3) mature and easily implementable preparation processes. Table 6 summarizes commonly used surface-layer materials for optical mirrors and their respective CTE [108].

For metallic mirrors such as aluminum alloys, NiP or Si are typically adopted as surface modification materials. NiP is a high-phosphorus amorphous coating capable of being deposited to thicknesses of hundreds of micrometers on aluminum alloy substrates. Its primary objective is to provide a dense, defect-free layer that facilitates subsequent SPDT and ultra-precision polishing. This approach effectively resolves the issues of grain boundaries and pitting anomalies typically encountered during the direct polishing of aluminum alloys, resulting in a significantly enhanced surface roughness [106]. However, owing to the substantial disparity in the linear CTE between the NiP plating and the AlSi10Mg substrate, the mirror experiences severe thermal deformation of its surface figure under significant temperature fluctuations. This phenomenon, known as the bimetallic bending effect, restricts its application in space environments characterized by wide temperature ranges.

To resolve this CTE mismatch issue, Tan et al. [79] selected pure Al as the surface modification material for AlSi10Mg mirrors. Experimental measurements demonstrated that over a temperature variation range of 100 K, the RMS surface figure change of the pure Al-modified mirror was merely 15.82 nm. This vastly outperforms its NiP-modified counterparts, thereby proving its exceptional thermal stability. Similarly, Chen [102] from CIOMP proposed a surface modification methodology utilizing nanosecond pulsed laser irradiation on the mirror surface. Through surface remelting and recrystallization, this technique effectively eradicates internal porosity and un-melted particles, forming a highly densified layer. Following nanosecond laser irradiation coupled with CMP, the final surface roughness can be refined to an impressive 3.7 nm (measured). In addition to altering the modification layer material or surface treatment processes as discussed above, researchers have also approached this issue from the substrate material perspective. By increasing the silicon content in the aluminum alloy, they developed a substrate system that matches the CTE of the NiP plating. For instance, Hilpert et al. [42] successfully demonstrated the additive manufacturing of metal mirrors using an aluminum-silicon alloy with 40 wt.% silicon (AlSi40). Utilizing AlSi40 as the substrate material significantly mitigates the thermal mismatch with the electroless NiP layer. This approach achieves an exceptionally thermally stable design, enabling the mirror to maintain superior optical performance across a broad temperature range from −185 °C to 100 °C.

For non-metallic mirrors such as SiC, commonly used surface modification materials include Si and SiC, which are typically deposited via CVD or PVD processes. Among these, CVD SiC has been widely applied for the surface modification of SiC substrates. For instance, Johnson [109] proposed a method for depositing an additional SiC layer on figured RB-SiC; Zhou et al. [110] also fabricated a CVD SiC coating on a C/SiC composite substrate, achieving a measured surface roughness of 0.2 nm RMS. However, the CVD process is not only complex and limited in yield, but its high-temperature deposition environment is also prone to inducing thermal deformation of the SiC substrate, thereby degrading the mirror’s surface figure accuracy. Consequently, researchers have turned to pure Si as an alternative coating material due to its close coefficient of thermal expansion match with SiC, aiming for superior thermal compatibility and dimensional stability. Jalluri et al. [111] successfully deposited a pure Si coating with a thickness of 350–400 μm on a sintered SiC substrate, achieving a stable surface micro-roughness between 1.7 and 1.9 nm RMS (measured).

In contrast, PVD processes are favored by the optical manufacturing industry owing to their lower deposition temperatures and relative operational simplicity [112]. Song et al. [113] proposed a novel surface modification method for Cf/SiC composites. By employing a tape casting and reaction bonding method, they fabricated a highly dense Si/SiC coating with robust substrate adhesion, achieving an excellent surface roughness of 2.16 nm RMS (measured). Liu et al. [114] selected pure Si as the modification material, utilizing magnetron sputtering technology to deposit a 15 μm modification layer onto a 2 m-class RB-SiC substrate. This drastically reduced the measured surface roughness from 5.64 nm RMS to an impressive 0.78 nm. Furthermore, Xu et al. [115] applied surface modifications to AM SiC mirrors, obtaining a 10 μm thick pure Si layer that, post-polishing, achieved a surface roughness of 1.109 nm $R_{a}$ (measured).

In addition to single-material modification layers, a hybrid strategy combining transition-layer modification with optical reflective coatings has also demonstrated considerable application potential. Bai et al. [116] introduced Cr, Ge, and Ti transition layers onto unmodified SiC substrates by thermal evaporation, followed by the deposition of Ag reflective coatings, to investigate the effects of different interlayers on the SiC/Ag interface properties. The results showed that all three transition layers effectively improved the interfacial characteristics of the SiC surface and contributed to the filling of surface micropores. Among them, the Ge transition layer exhibited the best performance in reducing surface roughness and suppressing optical scattering. Zhang et al. [117] employed a pure Si interlayer between the SiC-based substrate and the Ag reflective coating. The Si interlayer improves interfacial wettability and adhesion and helps suppress elemental interdiffusion. More importantly, its coefficient of thermal expansion is much closer to that of the SiC-based substrate than that of Ag, allowing it to act as a transitional layer that partially mitigates the large thermal-expansion mismatch between the substrate and the Ag coating. Dense Si and Ag films deposited by physical vapor deposition ultimately produced an ultrasmooth optical surface with an RMS roughness of 19.62 nm and an average visible-band reflectance of 97.2%.

## 5. Challenges in AM Mirrors

After years of development, AM mirrors have achieved significant milestones. The application of TO, lattice, and Voronoi architectures has further enhanced structural lightweighting, thermal stability, and structural integration. However, to realize their large-scale engineering application in space optical systems, several critical technological challenges remain to be resolved:

(1) Underutilization of 3D spatial topological advantages: In current research, while TO and TPMS methods can effectively utilize 3D space, numerous configurations, such as lattice and Voronoi structures, exhibit insufficient variable-density optimization along the optical axis. Future research trajectories should focus on deeply integrating lattice or Voronoi architectures with advanced TO algorithms. This aims to construct opto-mechanically driven multi-scale variable-density structures to maximize global stiffness and effectively mitigate localized thermo-mechanical stresses.

(2) Micro-geometric defects in Voronoi structures: The algorithmic generation of Voronoi architectures inevitably produces exceedingly short geometric edges. When these short edges are expanded into 3D solid entities such as ribs or struts, they readily form micro-struts and excessively narrow intersecting ribs. This severely compromises the stiffness of the mirror structure and induces localized stress concentrations. Consequently, it is crucial to develop adaptive algorithms capable of precisely identifying and eliminating these singular features during the modeling phase.

(3) Contradictions between aperture limitations and forming equipment: Constrained by the build volumes of current commercial AM equipment, the monolithic printing of large-aperture mirrors faces immense challenges. In particular, the fabrication of metallic mirrors currently stagnates at the 300 mm aperture level. Although non-metallic materials such as SiC can be utilized to manufacture slightly larger mirrors, a significant gap remains compared to the meter-class large-aperture mirrors required for future applications.

(4) Deficiencies in as-built quality and holistic process chains: The precision of current as-built AM components generally remains at the micrometer level, accompanied by poor microstructural densification that readily induces internal micro-cracks and high porosity. For instance, titanium alloy mirror blanks fabricated via EBM by Herzog et al. [23] at the University of Arizona still exhibited pervasive porosity on the surface after grinding and polishing. This underscores that the entire AM optical manufacturing ecosystem—spanning from the synthesis of high-quality powder feedstocks and optimization of processing parameters to precision post-processing—demands synergistic breakthroughs from both academia and industry.

## 6. Development Trends and Future Prospects

With the advancement of the aerospace industry and the escalating technical requirements for space optical systems, a multitude of cutting-edge design and optimization concepts have been applied to the lightweight design of mirror structures. Reviewing the developmental trajectory of AM mirrors in recent years, irregular architectures—such as TO, lattices, Voronoi, and TPMS—have exhibited exceptional thermo-mechanical performance, substantially increasing the design degrees of freedom for mirrors. The further evolution of AM technologies provides a robust manufacturing guarantee for the physical realization of these complex, lightweight mirrors. Concurrently, the rapid proliferation of AI in recent years has endowed human optomechanical design with unprecedented imaginative scope. Consequently, future research and development of 3D-printed mirrors will likely converge upon the following distinct trajectories:

(1) Deep integration of TO with multi-scale lightweight design: TO is employed to determine the optimized macroscopic material distribution of the mirror, which is subsequently mapped and coupled with lattice, TPMS, and Voronoi architectures. By exploiting the design freedom offered by 3D configurations and bio-inspired structures, hierarchical variable-density lightweight architectures can be constructed. On this basis, additive manufacturing constraints, including minimum feature size, overhang angle, build orientation, and support accessibility, are explicitly incorporated into the optimization process to achieve a reasonable balance between structural performance and manufacturing feasibility, thereby obtaining optimized structures that combine lightweight performance with practical manufacturability [118,119].

(2) Perfecting the holistic manufacturing process chain for 3D-printed mirrors: Establishing a traceable and standardized manufacturing workflow encompassing design, fabrication, precision post-processing, assembly, experimental validation, and final metrological inspection. Constructing such a comprehensive and high-performance optical manufacturing ecosystem is the pivotal prerequisite for ensuring deterministic nanometer-level surface figure accuracy.

(3) Monolithic integrated design of optomechanical systems: Breaking through the assembly barriers of traditional components to explore the monolithic printing of adjacent mirrors, or of mirrors integrated with their backing support structures, as exemplified by the monolithic primary–tertiary mirror design proposed by Heidler et al. [93]. This integrated philosophy not only achieves drastic macroscopic lightweighting but also significantly minimizes the overall part count, simplifies assembly procedures, and eradicates alignment-induced stresses, establishing it as a crucial evolutionary direction.

(4) Self-supporting or support-free structural design: Currently, the vast majority of AM mirrors necessitate auxiliary support structures during fabrication. Although the surrounding powder provides partial geometrical support, metal L-PBF generally still requires dedicated support structures for part anchoring, heat dissipation, and distortion control. Therefore, developing topologies or lattice structures with inherent self-supporting characteristics not only bolsters overarching structural stiffness but also vastly simplifies the powder removal and post-processing phases, representing another urgent frontier actively pursued by researchers.

(5) AI/ML-driven intelligent structural design of mirrors: AI and ML provide promising approaches for accelerating the optimization of complex lightweight structures. For example, Cao et al. [120] developed an ML-based gradient anisotropic Voronoi porous (GAVP) optimization method that improved stress uniformity and mechanical performance. ML has also been applied to topology optimization for surrogate modeling, design-space reduction, and generative design [121]. However, supervised ML generally requires substantial training data, and data generation for three-dimensional multiphysics problems can involve considerable computational cost. Generalization and explicit treatment of manufacturing constraints also remain challenging. Physics-guided approaches, including adjoint-based data augmentation [122] and fabrication-aware generative models [123], offer potential solutions by reducing data requirements and incorporating design constraints. Although these methods have mainly been demonstrated in topology optimization and photonic inverse design, they provide useful methodological guidance for AM mirrors. Future AI-assisted mirror design should integrate thermo-mechanical deformation, surface-figure and imaging performance, dynamic stiffness, and AM constraints to enable physics-guided, manufacturing-aware, multi-objective optimization.

## 7. Conclusions

This paper systematically reviews the manufacturing technologies for AM space mirrors, encompassing fabrication processes, structural design strategies, and post-processing procedures. It also summarizes the mainstream substrate and surface modification materials currently used in AM mirrors. Categorized by lightweight design paradigms, this review synthesizes recent developments in AM space mirrors, covering conventional structural designs, TO, and complex architectures such as lattices, TPMS, and Voronoi structures. Finally, the key technological challenges currently limiting the development of AM mirrors are critically discussed, and future research directions are outlined. Overall, AM substantially expands the design space for lightweight mirror architectures and provides considerable potential for the development of high-performance space optical mirrors.

## Figures and Tables

**Table 1:** Comparison of key characteristics of AM technologies relevant to aerospace applications [8,9,10,11,12,13,14,15].

Technology Category	Typical Materials	Advantages	Disadvantages
SLS	SiC Ti Alloy Powder Al Alloy Powder, etc.	Direct forming Capable of producing complex cavities and hard-to-machine alloys	Low forming efficiency Partial material melting Poor surface quality & density
SLM	Ti Alloy Powder Al Alloy Powder Iron Alloy Powder, etc.	Direct forming High dimensional accuracy & density Material-efficient	Low forming efficiency Limited part size
EBM	Ti Alloy Powder	High energy utilization efficiency High vacuum protection Good mechanical properties and high material integrity	Build size limited by powder bed High cost Relatively high as-built surface roughness
DED	Ti Alloy Powder Nickel-Based Superalloys, etc.	High deposition rate Capable of printing extra-large parts Wide range of material applicability	Poor geometric accuracy and surface texture Potential issues with residual stress and deformation Requires post-processing
SLA	SiC	High precision Short process cycle	Requires post-formation densification sintering Prone to defects (pores, cracks, deformation)
DLP	SiC Si_3_N_4_ ZrO_2_ Al_2_O_3_, etc.	Capable of micron-scale structure fabrication Fast forming speed High precision and good surface quality	Requires debinding and sintering of green body Time-consuming Difficult to fabricate large-sized parts
MEX	SiC Al_2_O_3_ PLA Nylon, etc.	Simple operation of printing equipment Fast forming speed Wide variety of materials and low cost Multi-material printing	Low resolution and surface finish High post-processing risk Not suitable for manufacturing large parts Mechanical anisotropy
BJT	SiC Si Metal powders (e.g., 316L, pure Cu), etc.	Support-free Ultra-fast printing Low thermal stress	Low density Complex post-processing

**Table 2:** Comparative analysis of material properties for conventional mirrors [20,29,30].

Material	Density ρ $\left(g/{\mathrm{cm}}^{3}\right)$	Elastic Modulus *E* (GPa)	Specific Stiffness E/ρ (kN·m/g)	Poisson Ratio μ	Thermal Conductivity λ (W/(m·K))	Coefficient of Thermal Expansion α $\left(10^{-6}/K\right)$
SiC	3.21	420.00	130.84	0.25	120.00	2.50
ULE *	2.21	67.00–73.00	30.32–33.03	0.17	1.31	0.00±0.03
Zerodur	2.53	91.00	35.97	0.24	1.64	0.05
Fused silica	2.19	72.00	32.88	0.17	1.40	0.50
Al	2.70	68.00	25.19	0.33	167.00	22.50
Be	1.85	287.00	155.14	0.043	216.00	11.40

* The CTE of ULE glass is the mean value specified over 5–35 °C.

**Table 3:** Representative material properties under specified manufacturing states relevant to AM mirrors.

Material	Manufacturing/Post-Processing State	Density ρ $\left(g/{\mathrm{cm}}^{3}\right)$	Elastic Modulus *E* (GPa)	Specific Stiffness E/ρ (kN·m/g)	Poisson’s Ratio μ	Thermal Conductivity λ (W/(m·K))	Coefficient of Thermal Expansion α $\left(10^{-6}/K\right)$	Source
AlSi10Mg	LPBF; stress relieved at 270 °C for 2 h, furnace cooled	2.68 ^a^	71.10	26.53	0.34 ^c^	168.00 ± 4.00	NR	[36,37]
AlSi12	DMP/LPBF; as-built; no heat treatment	2.69 ^a^	70.00 ± 5.00 (XY); 65.00 ± 5.00 (Z) ^b^	26.07 (XY); 24.21 (Z)	NR	130.00–150.00 ^d^	20.00 ^d^	[38]
Ti-6Al-4V	LPBF; heat treated at 800 °C for 2 h in Ar	≥4.40	110.00	≤25.0	NR	NR	NR	[39]
Si/SiC composite	VPP → carbon precursor infiltration/pyrolysis → RMI	3.04	355.48	116.93	NR	NR	2.39	[21]
Glass filled nylon	SLS; no heat treatment reported	1.23–1.28	3.20	2.50–2.60	NR	NR	68.00	[40]

^a^ Theoretical density reported by the material supplier. ^b^ XY and Z denote the build orientations parallel and perpendicular to the build plane, respectively. ^c^ Calculated from the reported Young’s and shear moduli assuming isotropic linear elasticity. NR: not reported. Specific stiffness was calculated from the density and elastic modulus listed in the table. ^d^ Literature-based thermophysical value reported in the manufacturer’s datasheet rather than a measurement for the specified material state.

**Table 4:** Summary of technical specifications for AM mirror finite element models.

Sample	Institution	Mirror Material	Diameter	Predicted Surface Deformation RMS (FEA)	Lightweighting Ratio or Mass	Reference & Layout
1	Oak Ridge National Laboratory [31]	SiC	184 mm	0.9 nm	0.45 kg	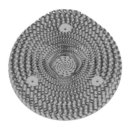
2	CIOMP [91]	AlSi10Mg	120 mm	19.02 nm (2.5D) 16.90 nm (3D)	156 g (2.5D) 163 g (3D)	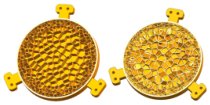
3	SITP [66]	SiC	510 mm (PM) 300 mm (QM)	11.81 nm (PM) 8.03 nm (QM)	5.18 kg	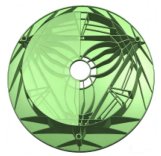
4	UESTC [86]	SiC	400 mm	<20 nm	70%	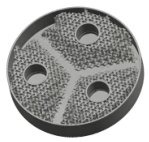
5	CIOMP [94]	AlSi10Mg	288 mm	2.82 nm	71.12%	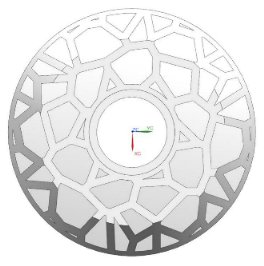

**Table 5:** Summary of measured performance and parameters for fabricated AM mirror prototypes.

Sample	Institution	Mirror Material	Manufacturing Method	Diameter	Surface Modification Layer	Measured Surface Figure Error RMS	Lightweighting Ratio or Mass	Reference & Layout
1	Aix-Marseille Université [24]	Al_2_O_3_	SLA	51 mm	NR	NR	NR	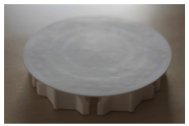
2	Oak Ridge National Laboratory [14]	SiC	BJ	270 mm	NR	9 nm	NR	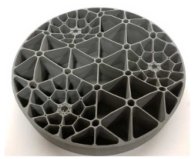
3	CIOMP [47]	AlSi10Mg	SLM	67 mm × 50 mm	NR	58.85 nm	43.7%	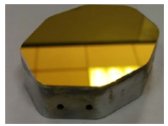
4	CIOMP [21]	SiC	SLA	500 mm	NR	NR	NR	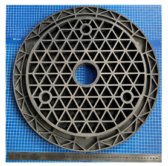
5	CIOMP [64]	AlSi10Mg	SLM	260 mm	NR	25.94 nm	1.11 kg	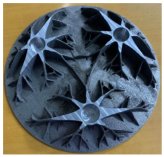
6	CIOMP [76]	AlSi10Mg	SLM	150 mm × 150 mm	NR	17.72 nm	85%	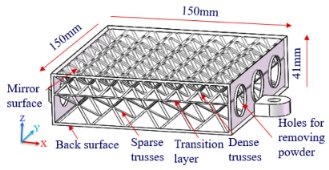
7	CIOMP [56]	AlSi10Mg	LPBF	175 mm (PM) 70 mm (TM)	NiP	64 nm (PM) 57 nm (TM)	NR	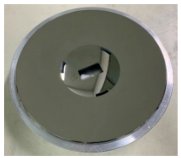
8	BIT [77]	AlSi10Mg	SLM	50 mm	NR	NR	20%	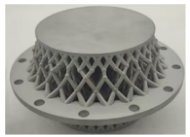
9	CIOMP [65]	AlSi10Mg	NR	58 mm	NiP	43.66 nm	36.3 g	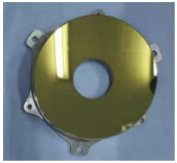
10	Sapienza University of Rome [78]	AA2024 RAM10	SLM	123.5 mm	NiP	NR	157.79 g	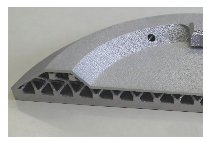
11	CIOMP [57]	AlSi10Mg	SLM	80 mm	NR	NR	NR	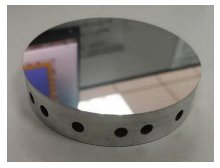
12	CIOMP [79]	AlSi10Mg	SLM	150 mm	Pure Al	12 nm	NR	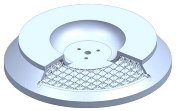
13	UK Astronomy Technology Centre [88]	AlSi10Mg	L-PBF	84 mm	NR	NR	56%	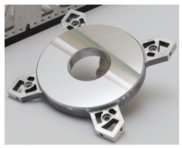
14	XIOPM [95]	AlSi10Mg	SLM	150 mm	NR	NR	0.366 kg	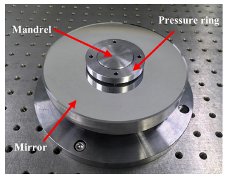

**Table 6:** Thermal expansion coefficients of representative substrates and surface layers [108].

Material	Al	NiP	Si	Ag
Coefficient of thermal expansion α ($10^{-6}$/K)	23.99	15	2.6	19.5
Functional role	Substrate /modification layer	Machinable modification layer	Transition /modification layer	Reflective optical coating

## Data Availability

No new data were created or analyzed in this study. Data sharing is not applicable to this article.

## Abbreviations

The following abbreviations are used in this manuscript:

AM	Additive manufacturing
TO	Topology optimization
ASTM	American Society for Testing and Materials
BJT	Binder jetting
DED	Directed energy deposition
MEX	Material extrusion
MJT	Material jetting
PBF	Powder bed fusion
SHL	Sheet lamination
VPP	Vat photopolymerization
SLA	Stereolithography
DLP	Digital light processing
SLM	Selective laser melting
SLS	Selective laser sintering
EBM	Electron beam melting
LoF	Lack-of-fusion
RMI	Reactive melt infiltration
RB-SiC	Reaction-bonded silicon carbide
LSI	Liquid silicon infiltration
CTE	Coefficient of thermal expansion
SPDT	Single-point diamond turning
CIOMP	Changchun Institute of Optics, Fine Mechanics and Physics
MRF	Magnetorheological finishing
MWIR/LWIR	Mid-wave and long-wave infrared
VIS/SWIR	Visible and short-wave infrared
DMLS	Direct metal laser sintering
PM	Primary mirror
TM	Tertiary mirror
SIMP	Solid Isotropic Material with Penalization
FEA	Finite element analysis
QM	Quaternary mirror
BCC	Body-centered cubic
FCC	Face-centered cubic
MIST	Moving iso-surface threshold
BIT	Beijing Institute of Technology
SDG	size and diameter gradient
TPMS	Triply periodic minimal surfaces
UESTC	University of Electronic Science and Technology of China
UK ATC	UK Astronomy Technology Centre
HIP	Hot isostatic pressing
IOF	Fraunhofer Institute for Applied Optics and Precision Engineering
TMA	Three-mirror-anastigmat
XIOMP	Xi’an Institute of Optics and Precision Mechanics
AI	Artificial intelligence
ML	Machine learning
LoF	Lack of fusion
KH	Keyholes
GBs	Gas-entrapped bubbles
DCR	Deep cold rolling
CHP	Controlled hammer peening
NDT	Non-destructive testing
XCT	X-ray computed tomography
CMP	Chemical mechanical polishing
SLP	Stress lap polishing
ABP	Airbag polishing
IBF	Ion beam finishing
SSD	Subsurface damage
IBS	Ion beam sputtering
SP	Smoothing polishing
CVD	Chemical vapor deposition
PVD	Physical vapor deposition
GAVP	Gradient anisotropic Voronoi porous

## References

[1] Liu R., Li Z., Xu W., Yang X., Zhang D., Yao Z., Yang K. (2021). Dynamic analysis and experiment of a space mirror based on a linear state space expression. Appl. Sci..

[2] An L., Zhang S., Zhou C., Jia J. (2019). Study on lightweight design for spatial mirror structure. Manuf. Autom..

[3] Chen Y., Huang B., You Z., Chan C., Huang T. (2016). Optimization of lightweight structure and supporting bipod flexure for a space mirror. Appl. Opt..

[4] Zhang K., Qu H., Guan H., Zhang J., Zhang X., Xie X., Yan L., Wang C. (2021). Design and Fabrication Technology of Metal Mirrors Based on Additive Manufacturing: A Review. Appl. Sci..

[5] Zhang C., Li Z. (2022). A Review of Lightweight Design for Space Mirror Core Structure: Tradition and Future. Machines.

[6] Heinrich A. (2021). 3D Printing of Optical Components.

[7] (2021). Additive Manufacturing—General Principles—Fundamentals and Vocabulary.

[8] Wang Y., Fu J., Liu W., Liang Y., Zhang M., Yin S. (2026). Application and development of additive manufacturing technology in the aerospace field. Mech. Electr. Eng. Technol..

[9] Zhou X., Li Q., Zhang T., Yue X., Liang J., Li J. (2026). Research progress in defect analysis and regulation mechanism of ceramic cores in stereolithographic additive manufacturing. J. Mater. Eng..

[10] Luo Z., Zhang X., Wu J., Guo L., Shi Y. (2026). Slurry preparation and forming process optimization of silicon nitride ceramic with digital light processing technology. J. Mater. Eng..

[11] Zhang X., Fu P., Zhao W., Wu J., Zheng B., Xu Z. (2025). Simulation and experimental study of a dual-light source co-curing system for DLP 3D printing of large-size ceramic materials. Ceramics.

[12] Goodman W.A., Nejhad M.N.G., Minei B.M., Stuecker J.N., Anderson T.M., Kroedel M., Goodman B.A. (2019). Ultra-lightweight ultra-stable RoboSiC additively manufactured lasercom telescope. Proceedings of the Material Technologies and Applications to Optics, Structures, Components, and Sub-Systems IV.

[13] Goodman B., Kim D.W., Rascher R. (2020). The results of Goodman Technologies NASA Phase II SBIRs for additive manufacturing of mirrors and telescopes. Proceedings of the Optical Manufacturing and Testing XIII.

[14] Horvath N., Honeycutt A., Davies M.A. (2020). Grinding of additively manufactured silicon carbide surfaces for optical applications. CIRP Ann..

[15] Atkins C., Chahid Y., Lister G., Tuck R., Isherwood D., Yu N., Sun R., Noto I., Yamamura K., Civitani M., Navarro R., Jedamzik R. (2024). Additive manufacturing in ceramics: Targeting lightweight mirror applications in the visible, ultraviolet, and x-ray. Proceedings of the Advances in Optical and Mechanical Technologies for Telescopes and Instrumentation VI.

[16] Mazzoli A. (2013). Selective laser sintering in biomedical engineering. Med. Biol. Eng. Comput..

[17] Zhang J., Song B., Wei Q., Bourell D., Shi Y. (2019). A review of selective laser melting of aluminum alloys: Processing, microstructure, property and developing trends. J. Mater. Sci. Technol..

[18] Bove A., Calignano F., Galati M., Iuliano L. (2022). Photopolymerization of Ceramic Resins by Stereolithography Process: A Review. Appl. Sci..

[19] Chaudhary R., Fabbri P., Leoni E., Mazzanti F., Akbari R., Antonini C. (2023). Additive manufacturing by digital light processing: A review. Prog. Addit. Manuf..

[20] Li W., Zhang G., Cui C., Bao J., Guo C. (2024). Research progress of additive manufacturing SiC ceramic mirror. Bull. Chin. Ceram. Soc..

[21] Li W., Cui C., Li S., Zhang G., Jin B., Bao J., Guo C., Zhang Y., Liu B., Wang G. (2024). Vat photopolymerization of large-aperture high performance SiC mirror through multiphase carbon infiltration modification. Addit. Manuf..

[22] Murr L.E., Gaytan S.M., Ramirez D.A., Martinez E., Hernandez J., Amato K.N., Shindo P.W., Medina F.R., Wicker R.B. (2012). Metal Fabrication by Additive Manufacturing Using Laser and Electron Beam Melting Technologies. J. Mater. Sci. Technol..

[23] Herzog H., Segal J., Smith J., Bates R., Calis J., Torre A.D.L., Kim D.W., Mici J., Mireles J., Stubbs D.M., Hatheway A.E. (2015). Optical fabrication of lightweighted 3D printed mirrors. Proceedings of the Optomechanical Engineering 2015.

[24] Roulet M., Atkins C., Hugot E., Snell R., van de Vorst B., Morris K., Marcos M., Todd I., Miller C., Dufils J., Herkommer A.M., von Freymann G., Flury M. (2020). Use of 3D printing in astronomical mirror fabrication. Proceedings of the 3D Printed Optics and Additive Photonic Manufacturing II.

[25] Atkins C., Brzozowski W., Dobson N., Milanova M., Todd S., Pearson D., Bourgenot C., Brooks D., Snell R., Sun W., Hull T.B., Kim D.W., Hallibert P. (2019). Additively manufactured mirrors for CubeSats. Proceedings of the Astronomical Optics: Design, Manufacture, and Test of Space and Ground Systems II.

[26] Xue Y., Li Z., Fu X. (2025). Research progress on the application of 3D printing technology in energetic materials. Chin. J. Explos. Propellants.

[27] Li T., Zhang Y., Gong X., Liu K., Yang M., Zhang S., Han X., Yang L., Yan C., Shi Y. (2024). Research status and prospects of 3D printing of fiber-reinforced silicon carbide ceramic matrix composites. J. Netshape Form. Eng..

[28] Zhang W., Zhang G., Guo C., Fan T., Xu C. (2020). Fiber-reinforced silicon carbide and its applications in optical mirrors. Chin. Opt..

[29] Yu L., Guan M., Han X., Zhao C., Zhu Y. (2020). Research progress of lightweight mirror materials in space remote sensor. China Build. Mater. Sci. Technol..

[30] Guo S., Wang W., Zhang G., Zhao X. (2005). Lightweight mirror technology for space optical systems. Opt. Instrum..

[31] Horvath N., Davies M. (2021). Advancing lightweight mirror design: A paradigm shift in mirror preforms by utilizing design for additive manufacturing. Appl. Opt..

[32] Hu H., Zhao H., Liu Z., Luo X., Zhang X. (2017). Hydrostatic support system for in-situ optical testing of a 4 m aperture SiC mirror. Opt. Precis. Eng..

[33] Zhang X., Hu H., Wang X., Luo X., Zhang G., Zhao W., Wang X., Liu Z., Xiong L., Qi E. (2022). Challenges and strategies in high-accuracy manufacturing of the world’s largest SiC aspheric mirror. Light. Sci. Appl..

[34] Li K. (2023). SLM Forming and Performance Regulation of (TiB_2_+SiC)/AlSi10Mg Composite for Mirrors. Ph.D. Thesis.

[35] Paenoi J., Bourgenot C., Atkins C., Snell R., Todd I., White P., Parkin K., Ryder D., Kotlewski R., McPhee S. (2022). Lightweight, aluminum, mirror design optimization for conventional and additive manufacturing processes. Proceedings of the Advances in Optical and Mechanical Technologies for Telescopes and Instrumentation V.

[36] Van Cauwenbergh P., Samaee V., Thijs L., Nejezchlebová J., Sedlák P., Iveković A., Schryvers D., Van Hooreweder B., Vanmeensel K. (2021). Unravelling the multi-scale structure–property relationship of laser powder bed fusion processed and heat-treated AlSi10Mg. Sci. Rep..

[37] 3D Systems, Inc. (2023). LaserForm AlSi10Mg (A): Material Datasheet.

[38] 3D Systems, Inc. (2018). LaserForm AlSi12 (B): Material Datasheet.

[39] EOS GmbH (2022). EOS Titanium Ti64: Material Data Sheet.

[40] Atkins C., Feldman C., Brooks D., Watson S., Cochrane W., Roulet M., Doel P., Willingale R., Hugot E., O’Dell S.L., Pareschi G. (2017). Additive manufactured X-ray optics for astronomy. Proceedings of the Optics for EUV, X-Ray, and Gamma-Ray Astronomy VIII.

[41] Miao Q., Liu M., Zhao K., Ma G., Wu D. (2018). Research progress on technologies of additive manufacturing of aluminum alloys. Laser Optoelectron. Prog..

[42] Hilpert E., Hartung J., von Lukowicz H., Herffurth T., Heidler N. (2019). Design, additive manufacturing, processing, and characterization of metal mirror made of aluminum silicon alloy for space applications. Opt. Eng..

[43] Chen Z., Qu P., Ji Z. (2025). Research progress on additive manufacturing for SiC-based ceramics and its strengthening and toughening. Aeronaut. Manuf. Technol..

[44] Li S., Zhang H., Han Y., Lu Z., Miao K., Wang Z., Li D. (2023). Thermally assisted extrusion-based 3D printing of continuous carbon fiber-reinforced SiC composites. Compos. Part A Appl. Sci. Manuf..

[45] Zhang K., Chen Y., Weng L., Cheng S., Yu S., Zeng T. (2024). Fabrication of dense Cf/SiC ceramic matrix composites using 3D printing and PIP with short carbon fibre as a toughening material. Virtual Phys. Prototyp..

[46] Wang W., Gao X., Li Z., Shen C., He R. (2024). Effects of ceramic layer height on the mechanical properties of Cf/SiC ceramic matrix composites fabricated by fiber-laying-assisted material extrusion 3D printing. Compos. Commun..

[47] Tan S., Ding Y., Xu Y., Shi L. (2020). Design and fabrication of additively manufactured aluminum mirrors. Opt. Eng..

[48] Tan S., Ding Y., Xu Y., Liu W. (2020). Development of additively manufacturing metal mirrors. Chin. Opt..

[49] Atkins C., van de Vorst L.G.T.B., Conley A., Farkas S., Hugot E., Mező G., Morris K., Roulet M., Snell R.M., Tenegi-Sanginés F., Navarro R., Geyl R. (2022). The OPTICON A2IM Cookbook: An introduction to additive manufacture for astronomy. Proceedings of the Advances in Optical and Mechanical Technologies for Telescopes and Instrumentation V.

[50] Li W., Cui C., Bao J., Zhang G., Li S., Wang G. (2021). Properties regulation of SiC ceramics prepared via stereolithography combined with reactive melt infiltration techniques. Ceram. Int..

[51] Valente T.M., Vukobratovich D., Vukobratovich D. (1989). A Comparison of the Merits of Open-Back, Symmetric Sandwich, and Contoured Back Mirrors as Light-Weighted Optics. Proceedings of the Precision Engineering and Optomechanics.

[52] Qu Y., Jiang Y., Feng L., Li X., Liu B., Wang W. (2018). Lightweight Design of Multi-Objective Topology for a Large-Aperture Space Mirror. Appl. Sci..

[53] Woodard K.S., Comstock L.E., Wamboldt L., Sutherland J.S., Vizgaitis J.N., Andresen B.F., Marasco P.L., Sanghera J.S., Snyder M.P. (2016). Optimum selection of high performance mirror substrates for diamond finishing. Proceedings of the Advanced Optics for Defense Applications: UV Through LWIR.

[54] Sweeney M., Acreman M., Vettese T., Myatt R., Thompson M., Krödel M., Robichaud J.L., Goodman W.A. (2015). Application and testing of additive manufacturing for mirrors and precision structures. Proceedings of the Material Technologies and Applications to Optics, Structures, Components, and Sub-Systems II.

[55] Hilpert E., Hartung J., Risse S., Eberhardt R., Tünnermann A. (2018). Precision manufacturing of a lightweight mirror body made by selective laser melting. Precis. Eng..

[56] Zhang J., Wang C., Qu H., Guan H., Wang H., Zhang X., Xie X., Wang H., Zhang K., Li L. (2022). Design and Fabrication of an Additively Manufactured Aluminum Mirror with Compound Surfaces. Materials.

[57] Tan S., Xu Y., Wang Y., Shi L., Masrour R., Xu X. (2024). Optical surface roughness improvement using hot isostatic pressing and coating of additively manufactured mirrors. Proceedings of the International Conference on Optoelectronic Information and Functional Materials (OIFM 2024).

[58] Sun Y., Fan Y., Dong D., Li C., Jiang X. (2022). A review of space mirror topology optimization technology based on additive manufacturing. Laser J..

[59] Liu G., Guo L., Wang X., Wu Q. (2018). Topology and parametric optimization based lightweight design of a space reflective mirror. Opt. Eng..

[60] Bendsøe M., Sigmund O. (1999). Material interpolation schemes in topology optimization. Arch. Appl. Mech..

[61] Amstutz S. (2011). Connections between topological sensitivity analysis and material interpolation schemes in topology optimization. Struct. Multidiscip. Optim..

[62] Liu S., Hu R., Li Q., Zhou P., Dong Z., Kang R. (2014). Topology optimization-based lightweight primary mirror design of a large-aperture space telescope. Appl. Opt..

[63] Atkins C., Feldman C., Brooks D., Watson S., Cochrane W., Roulet M., Hugot E., Beardsley M., Harris M., Spindloe C., Navarro R., Geyl R. (2018). Topological design of lightweight additively manufactured mirrors for space. Proceedings of the Advances in Optical and Mechanical Technologies for Telescopes and Instrumentation III.

[64] Fan Y., Dong D., Li C., Sun Y., Zhang Z., Wu F., Yang L., Li Q., Guan Y. (2021). Research and Experimental Verification on Topology-Optimization Design Method of Space Mirror Based on Additive-Manufacturing Technology. Machines.

[65] Yan L., Zhang X., Fu Q., Wang L., Shi G., Tan S., Zhang K., Liu M. (2022). Assembly-level topology optimization and additive manufacturing of aluminum alloy primary mirrors. Opt. Express.

[66] Shen X., Sun X., Wang C., Yang Y., Sun L., Chen J., Sun S. (2024). Topology optimization of a double-sided space mirror based on additive manufacturing of SiC. Struct. Multidiscip. Optim..

[67] Dong G., Tang Y., Zhao Y.F. (2017). A Survey of Modeling of Lattice Structures Fabricated by Additive Manufacturing. J. Mech. Des..

[68] Panesar A., Abdi M., Hickman D., Ashcroft I. (2018). Strategies for functionally graded lattice structures derived using topology optimisation for Additive Manufacturing. Addit. Manuf..

[69] Li C., Lei H., Zhang Z., Zhang X., Zhou H., Wang P., Fang D. (2020). Architecture design of periodic truss-lattice cells for additive manufacturing. Addit. Manuf..

[70] Tian L., Lv L., Peng H., Liu P., Yan X., Liu L., Tu C. (2023). A review of single-material geometric microstructure design. Chin. J. Comput..

[71] Deshpande V., Fleck N., Ashby M. (2001). Effective properties of the octet-truss lattice material. J. Mech. Phys. Solids.

[72] Deshpande V.S., Ashby M.F., Fleck N.A. (2001). Foam topology: Bending versus stretching dominated architectures. Acta Mater..

[73] Atkins C., Brzozowski W., Dobson N., Milanova M., Todd S., Pearson D., Bourgenot C., Brooks D., Snell R., Sun W., Hull T.B., Kim D.W., Hallibert P. (2019). Lightweighting design optimisation for additively manufactured mirrors. Proceedings of the Astronomical Optics: Design, Manufacture, and Test of Space and Ground Systems II.

[74] Snell R., Atkins C., Schnetler H., Todd I., Hernández-Nava E., Lyle A.R., Maddison G., Morris K., Miller C., Roulet M., Navarro R., Geyl R. (2020). An additive manufactured CubeSat mirror incorporating a novel circular lattice. Proceedings of the Advances in Optical and Mechanical Technologies for Telescopes and Instrumentation IV.

[75] Zhang M., Su Y., Wang C. (2021). A Non-uniform Lattice Structure Size Optimization Design Based on Space Mirror. Spacecr. Recovery Remote Sens..

[76] Liu C., Xu K., Zhang Y., Hu H., Tao X., Zhang Z., Deng W., Zhang X. (2022). Design and Fabrication of Extremely Lightweight Truss-Structured Metal Mirrors. Materials.

[77] Yang D., Pan C., Zhou Y., Han Y. (2022). Optimized design and additive manufacture of double-sided metal mirror with self-supporting lattice structure. Mater. Des..

[78] Boschetto A., Bottini L., Macera L. (2023). Design and fabrication by selective laser melting of a LIDAR reflective unit using metal matrix composite material. Int. J. Adv. Manuf. Technol..

[79] Tan S., Xu Y., Wang Y., Li H. (2025). Research on pure al modified layer for additively manufactured mirrors. J. Phys. Conf. Ser..

[80] Yoo D. (2011). Computer-aided porous scaffold design for tissue engineering using triply periodic minimal surfaces. Int. J. Precis. Eng. Manuf..

[81] Chen S. (2021). Design and Optimization of 3D Printed Objects Lightweight Using Triply Periodic Minimal Surface. Master’s Thesis.

[82] Yu X., Li L., Zhang T., Zhang Z. (2022). Modeling of complex contour lattice structure based on triply periodic minimal surface and voxel distance field. J. Chongqing Univ..

[83] Yan X. (2022). Efficient Modeling and Manufacturing of Complex Structures for Additive Manufacturing. Ph.D. Thesis.

[84] Liu F., Mao Z., Zhang P., Zhang D.Z., Jiang J., Ma Z. (2018). Functionally graded porous scaffolds in multiple patterns: New design method, physical and mechanical properties. Mater. Des..

[85] Zhou H. (2020). Research on Mechanical Properties and Topology Optimization Application of Gradient Lattice Structures for Additive Manufacturing. Master’s Thesis.

[86] Zeng C., Wang W., Hai K., Ma S. (2024). Lightweight airborne TPMS-filled reflective mirror design for low thermal deformation. Compos. Struct..

[87] Lister G., Tuck R., Chahid Y., Morris K., Kotlewski R., McPhee S., Bourgenot C., Parkin K., Beardsley M., Civitani M., Navarro R., Jedamzik R. (2024). Design, manufacture, and metrology of additively manufactured, metal, and ceramic lightweight circular mirror prototypes. Proceedings of the Advances in Optical and Mechanical Technologies for Telescopes and Instrumentation VI.

[88] Aziz I., Chahid Y., Keogh J., Carruthers J., Morris K., Harman J., McPhee S., Fraser E., Millan L., Bourgenot C., Hull T.B., Kim D., Hallibert P. (2025). Additive manufacturing in aluminium of a primary mirror for a CubeSat application: Manufacture, testing, and evaluation. Proceedings of the Astronomical Optics: Design, Manufacture, and Test of Space and Ground Systems V.

[89] Okabe A., Boots B., Sugihara K. (1994). Nearest neighbourhood operations with generalized Voronoi diagrams: A review. Int. J. Geogr. Inf. Syst..

[90] Berrocal L., Fernández R., González S., Periñán A., Tudela S., Vilanova J., Rubio L., Martín Márquez J.M., Guerrero J., Lasagni F. (2019). Topology optimization and additive manufacturing for aerospace components. Prog. Addit. Manuf..

[91] Zhang K., Xie X., Wang C., Wang H., Xu F., Wang H., Zhang X., Guan H., Qu H., Zhang J. (2022). Optomechanical Performances of Advanced Lightweight Mirrors Based on Additive Manufacturing. Micromachines.

[92] Liu B., Cai L., Cao W., Lu P. (2025). A stress-driven bi-level design method for variable radius Voronoi porous structures with enhanced mechanical performance. Comput. Methods Appl. Mech. Eng..

[93] Heidler N., Hilpert E., Hartung J., von Lukowicz H., Damm C., Peschel T., Risse S., Schröder S., Geyl R. (2018). Additive manufacturing of metal mirrors for TMA telescope. Proceedings of the Optical Fabrication, Testing, and Metrology VI.

[94] Zhang C., Li Z., Tao S., Li Q., Zhao Y., Ren S., Li Y., Ma B., Yu M. (2024). Optimal design and analysis of a space lightweight mirror with a directionally oriented 2.5D-CVT structure. Appl. Opt..

[95] Chen W., Li S., Sun L., Zhu Y., Long J., Wang Y., Hu B., Wang S. (2025). Design method for bionic topology optimization of high-specific-stiffness mirrors based on additive manufacturing. Appl. Opt..

[96] Mici J., Rothenberg B., Brisson E., Wicks S., Stubbs D.M., Hatheway A.E. (2015). Optomechanical performance of 3D-printed mirrors with embedded cooling channels and substructures. Proceedings of the Optomechanical Engineering 2015.

[97] Snell R., Atkins C., Schnetler H., Chahid Y., Beardsley M., Harris M., Zhang C., Pears R., Thomas B., Saunders H., Navarro R., Geyl R. (2022). Towards understanding and eliminating defects in additively manufactured CubeSat mirrors. Proceedings of the Advances in Optical and Mechanical Technologies for Telescopes and Instrumentation V.

[98] Tan S., Li Q., Xu Y., Shen H., Cheng Y., Jia P., Xu Y. (2022). Design and fabrication of lightweight additively manufactured mirrors for aviation. Appl. Opt..

[99] Chan C., Choi J., Hall C., James A., Smith C., Stephenson P.C.L., Zuber K. (2026). Heat treatment and precision machining of additively manufactured complex mirror substrates for compact optical payloads. Opt. Mater..

[100] Wang Y., Yu J., Sheng P., Xia J., Zhu D., Gu W., Xian R., Liu X., Wang D., Han F. (2025). Effect of pre-heat treatment on the inherent feature and diamond machined surface morphologies of AlSi10Mg manufactured by laser powder bed fusion. Mater. Today Commun..

[101] Maharjan N., Bhowmik A., Kum C., Hu J., Yang Y., Zhou W. (2021). Post-Processing of Cold Sprayed Ti-6Al-4V Coatings by Mechanical Peening. Metals.

[102] Chen L. (2023). Research on Surface Processing and Stabilization Treatment Technology of Additively Manufactured Truss-Structure Mirrors. Ph.D. Thesis.

[103] Du C., Dai Y., Guan C., Hu H. (2021). High efficiency removal of single point diamond turning marks on aluminum surface by combination of ion beam sputtering and smoothing polishing. Opt. Express.

[104] Tao M. (2024). Research on Magnetorheological Ultra-Precision Polishing Technology of NiP Coating on Aluminum Alloy Mirrors. Ph.D. Thesis.

[105] Bai Y., Zhang Z., Li L., Luo X., Li F., Zhang X. (2024). High precision polishing of aluminum alloy mirrors through a combination of magnetorheological finishing and chemical mechanical polishing. Opt. Express.

[106] Pradhan P., Shurvinton R., Bourgenot C., Wang H., Sawhney K. (2025). Ultra-high precision NiP mirror fabrication using ion beam figuring for space applications. Opt. Express.

[107] Ali M., Almotari A., Algamal A., Qattawi A. (2023). Recent Advancements in Post Processing of Additively Manufactured Metals Using Laser Polishing. J. Manuf. Mater. Process..

[108] Tan S. (2022). Research on Key Technologies of Metal Mirrors Based on Additive Manufacturing. Ph.D. Thesis.

[109] Johnson S.G., Wilkerson G.W. (1993). SiC coatings on RB SiC mirrors for ultrasmooth surfaces. Proceedings of the Passive Materials for Optical Elements II.

[110] Zhou H., Zhang C., Cao Y., Zhou X., Zhang Y., Jiang W., Cho M.K. (2006). Lightweight C/SiC mirrors for space application. Proceedings of the 2nd International Symposium on Advanced Optical Manufacturing and Testing Technologies: Large Mirrors and Telescopes.

[111] Jalluri T.D.P.V., Somashekar S., Dey A., Venkateswaran R., Elumalai S., Rudraswamy B., Sriram K.V. (2021). Characterization of thermal sprayed Si on sintered SiC for space optical applications. Surf. Eng..

[112] Jiang F., Liu Y., Yang Y., Huang Z.R., Li D., Liu G.l., Liu X.J. (2012). Research Progress of Optical Fabrication and Surface-Microstructure Modification of SiC. J. Nanomater..

[113] Song S., Yin J., Li Y., Zhu Y., Huang Y., Cheng G., Liu X., Huang Z. (2016). Si/SiC coated Cf/SiC composites via tape casting and reaction bonding: The effect of carbon content. Ceram. Int..

[114] Liu Z., Gao J., Liu H., Wang X., Wang T. (2016). Surface modification of 2m RB-SiC substrate by magnetron sputtering. Opt. Precis. Eng..

[115] Xu C., Hu H., Peng X., Lai T., Bao J., Li X., Duan X.M., Pu M., Wang C., Hu S., Luo X. (2021). Ultra-precision manufacturing of silicon carbide mirrors with additive manufacturing. Proceedings of the 10th International Symposium on Advanced Optical Manufacturing and Testing Technologies: Advanced and Extreme Micro-Nano Manufacturing Technologies.

[116] Bai Y., Wang L., Wang G., Zhang J., Zhou Y., Chu J., Shao J. (2019). Influence of transition layer on the surface scattering of unmodified silicon carbide. Proceedings of the Tenth International Conference on Thin Film Physics and Applications (TFPA 2019).

[117] Zhang B., Wang L., Chen X., Wei Y., Tian H., Huang Z., Liu X., Chen Z., Yin J. (2026). From Complex Shaping to Mirror Finish: Additive Manufacturing of Aerospace-grade Cf/SiC Space Optics. Adv. Sci..

[118] Langelaar M. (2017). An additive manufacturing filter for topology optimization of print-ready designs. Struct. Multidiscip. Optim..

[119] Thore C.-J., Grundström H.A., Torstenfelt B., Klarbring A. (2019). Penalty regulation of overhang in topology optimization for additive manufacturing. Struct. Multidiscip. Optim..

[120] Cao W., Lin H., Liu B., Lu P., Shen W., Huang J. (2026). Optimization of gradient anisotropic voronoi porous structures based on machine learning. Eng. Struct..

[121] Shin S., Shin D., Kang N. (2023). Topology optimization via machine learning and deep learning: A review. J. Comput. Des. Eng..

[122] Kang C., Seo D., Boriskina S.V., Chung H. (2024). Adjoint method in machine learning: A pathway to efficient inverse design of photonic devices. Mater. Des..

[123] Seo D., Um S., Lee S., Ye J.C., Chung H. (2026). Physics-guided and fabrication-aware inverse design of photonic devices using diffusion models. ACS Photonics.

